# OsNAC2 integrates auxin and cytokinin pathways to modulate rice root development

**DOI:** 10.1111/pbi.13209

**Published:** 2019-08-07

**Authors:** Chanjuan Mao, Jianmei He, Lina Liu, Qiming Deng, Xuefeng Yao, Chunming Liu, Yongli Qiao, Peng Li, Feng Ming

**Affiliations:** ^1^ Shanghai Key Laboratory of Plant Molecular Sciences College of Life Sciences Shanghai Normal University Shanghai China; ^2^ State Key Laboratory of Genetic Engineering Institute of Genetics Institute of Plant Biology School of Life Sciences Fudan University Shanghai China; ^3^ Institute of Rice Research Sichuan Agricultural University Chengdu China; ^4^ Key Laboratory of Plant Molecular Physiology Institute of Botany Chinese Academy of Sciences Beijing China; ^5^ Institute of Crop Sciences Chinese Academy of Agricultural Sciences Beijing China; ^6^ The Biotechnology Research Institute Shanghai Academy of Agricultural Sciences Shanghai China

**Keywords:** rice, root development, OsNAC2, auxin, cytokinin

## Abstract

The rice root system is important for growth. The crosstalk between auxin and cytokinin mediates root initiation and elongation. However, it remains unclear how the transcriptional network upstream of the auxin and cytokinin signalling pathways determines root development. Here, we observed that the knockdown of *OsNAC2*, which encodes a NAC transcription factor, increased the primary root length and the number of crown roots. *OsNAC2* predominantly expressed in primary root tips, crown roots and lateral root primordia, implying it influences root development. Molecular analyses revealed that the expressions of auxin‐ and cytokinin‐responsive genes were affected in *OsNAC2*‐overexpressing (*OsNAC2‐OX*; *ON7* and *ON11*), RNA interference (*OsNAC2‐RNAi*; *RNAi25* and *RNAi31*) and CRISPR/Cas9 plants. Additionally, OsNAC2 can directly bind to the promoters of IAA inactivation‐related genes (*GH3.6* and *GH3.8*), an IAA signalling‐related gene (*OsARF25*), and a cytokinin oxidase gene (*OsCKX4*). Furthermore, genetic analysis of *ON11*/*osgh3.6* and *RNAi31*/*osckx4* homozygote confirmed that OsCKX4 and OsGH3.6 functioned downstream of OsNAC2. The mRNA levels of CROWN ROOTLESS (CRL) genes and cyclin‐dependent protein kinase (CDK) genes increased in *OsNAC2‐RNAi* and *OsNAC2‐cas9* lines while reduced in *OsNAC2‐OX* lines. Thus, we describe that OsNAC2 functions as an upstream integrator of auxin and cytokinin signals that affect CRL and CDK production to regulate cell division during root development. This novel auxin‐OsNAC2‐cytokinin model should provide a new insight into the understanding of NAC TFs and crosstalk of auxin and cytokinin pathway, and can be potentially applied in agriculture to enhance rice yields by genetic approaches.

## Introduction

Rice (*Oryza sativa* L.) is one of the most important staple foods worldwide. The root system is important for the absorption of nutrients and water, anchoring of the plant and hormone biosynthesis, all of which are critical for crop growth and yield (Coudert *et al*., 2010). Optimizing root system architecture can overcome yield limitations in crop plants. As an important model monocot plant, the root system of rice is composed of a primary root, lateral roots and adventitious roots, which strikingly differs from that of *Arabidopsis thaliana*. Unlike that the regulatory mechanism associated with the *A. thaliana* root system has been well studied, the mechanism underlying rice root development remains largely unknown.

Many phytohormones, especially auxin and cytokinin, have been reported to function in root initiation and growth (Laplaze *et al*., 2007; Saini *et al*., 2013). For example, root morphological abnormalities were observed in a number of rice mutants defective in auxin biosynthesis or signalling. Mutations to the rice *COW1* gene, which belongs to the *YUCCA* gene family, result in the production of relatively few adventitious roots (Woo *et al*., 2007). Additionally, the indole‐3‐acetic acid (IAA)‐amido synthetase encoded by Gretchen Hagen 3 (GH3) affects auxin homeostasis by conjugating amino acids to IAA (Jain *et al*., 2006b). The ectopic expression of *OsGH3.2* in rice leads to an IAA‐deficient morphology, including the production of relatively few crown roots and root hairs (Du *et al*., 2012). Similarly, the *tld1‐D* (*osgh3.13*) mutant reportedly has fewer lateral and adventitious roots than the wild type (WT; Zhang *et al*., 2009). Furthermore, the ectopic expression of *OsAUX1*, which is related to polar auxin transport, enhances lateral root initiation (Yu *et al*., 2015). Moreover, rice *osiaa3* mutants are insensitive to auxin and gravitropic stimuli, with fewer crown roots than in normal rice plants (Nakamura *et al*., 2006). The root elongation zones of *osarf12* and *osarf12⁄25* mutants are significantly shorter than those of WT plants, probably because of a decrease in auxin synthesis and transport (Qi *et al*., 2012). Additionally, auxin promotes crown root initiation through the LOB‐domain transcription factor CROWN ROOTLESS1 (CRL1), and the *crl1/arl1* mutant produces only a few crown roots (Coudert *et al*., 2015). The same phenotype is observed in *crl4* mutants (Liu *et al*., 2009). The defective crown root of the *oscand1* mutant, a homolog of *AtCAND1*, is the result of a cessation of the cell cycle transition from the G2 stage to the M stage (Wang *et al*., 2011).

Cytokinin and auxin have an antagonistic relationship during root formation (Durbak *et al*., 2012). The overexpression of *OsIPT*, which encodes an isopentenyl transferase that catalyses the rate‐limiting step of cytokinin biosynthesis, inhibits root development (Sakamoto *et al*., 2006). Likewise, loss‐of‐function mutations to *LONELY GUY* (*LOG*), which encodes a cytokinin‐activating enzyme, retard root growth (Tokunaga *et al*., 2012). In contrast, transgenic plants overexpressing cytokinin oxidase/dehydrogenase (CKX) genes (e.g. *OsCKX4*) produce less than normal amounts of cytokinins and exhibit enhanced root growth and branching (Gao *et al*., 2014). Moreover, the knockdown or overexpression of rice genes involved in the cytokinin signalling pathway also affects root development. For example, the overexpression of *OsRR3* or *OsRR5* increases root length and the production of lateral roots (Cheng *et al*., 2010), while the ectopic expression of *OsRR2* promotes crown root initiation (Zhao *et al*., 2015). However, the molecular mechanism underlying auxin‐ and cytokinin‐regulated root development remains largely uncharacterized.

Available evidence suggests that the coordinated activities of auxin and cytokinin are crucial for root development (Ruzicka *et al*., 2009). In rice roots, the auxin‐induced expression of *OsCRL5* promotes crown root initiation by repressing cytokinin signalling, which positively regulates the expression of the type‐A RR protein‐encoding *OsRR1* gene (Kitomi *et al*., 2011a). Likewise, OsCKX4 positively regulates crown root formation targeted by the auxin response factor gene *OsARF25* and cytokinin RR genes, *OsRR2* and *OsRR3* (Gao *et al*., 2014). Moreover, the WUSCHEL‐related homeobox gene, *OsWOX11*, is important for integrating auxin and cytokinin signals, and directly represses the expression of a type‐A RR gene, *OsRR2*, during crown root development (Zhao *et al*., 2015). However, it is currently unclear how crosstalk between auxin and cytokinin regulates root initiation and elongation.

The plant‐specific NAC (NAM, ATAF and CUC) transcription factors have multiple roles related to plant growth and development. In *A. thaliana*,* AtNAC1* may promote lateral root formation by activating two downstream auxin‐responsive genes, *DBP* and *AIR3* (Xie *et al*., 2000). Additionally, *AtNAC2* may incorporate environmental and endogenous stimuli to enhance plant lateral root development (He *et al*., 2005). Meanwhile, *VND6* and *VND7* specifically improve metaxylem and protoxylem vessel formation in roots, and the SRDX lines (with a repression domain linked to the C‐terminal of transcription activators) produce primary roots that are abnormally short (Kubo *et al*., 2005). Transgenic *ATAF1*‐overexpressing lines reportedly exhibit dwarfism, with short primary roots (Wu *et al*., 2009). In contrast, the effects of NAC transcription factors on rice root development have not been fully investigated. The ectopic expression of *OsNAC10*,* OsNAC5* and *SNAC1* driven by a root‐specific promoter, RCc3, may enlarge the root diameter and improve drought resistance (Jeong *et al*., 2010, 2013; Redillas *et al*., 2012). However, there has been no detailed molecular characterization of any member of the NAC family regarding their effects on rice root development.

We previously reported that a rice NAC family transcription factor, OsNAC2, affects plant height (Chen *et al*., 2015) and regulates abiotic stress tolerance (Shen *et al*.,^2017^), senescence (Mao *et al*., 2017) and programmed cell death (Mao *et al*., 2018). In this study, we reveal that *OsNAC2*‐RNA interference and CRISPR/Cas9 plants exhibit enhanced root growth, while *OsNAC2*‐overexpression lines showed the opposite phenotype. Molecular and genetic analyses indicated that OsNAC2 functions as an upstream integrator of auxin and cytokinin signals. The coordinated action of these two hormones is essential for CRL and CDK production for maintaining root meristem size and ensuring root growth. The novel regulatory model involving auxin, NAC transcription factors and cytokinin herein provides new insights into the molecular action of phytohormones that modulate root formation, and has potential application in agriculture to enhance rice yields by genetic approaches.

## Results

### The expression pattern of *OsNAC2* during root development

**Figure 1:**
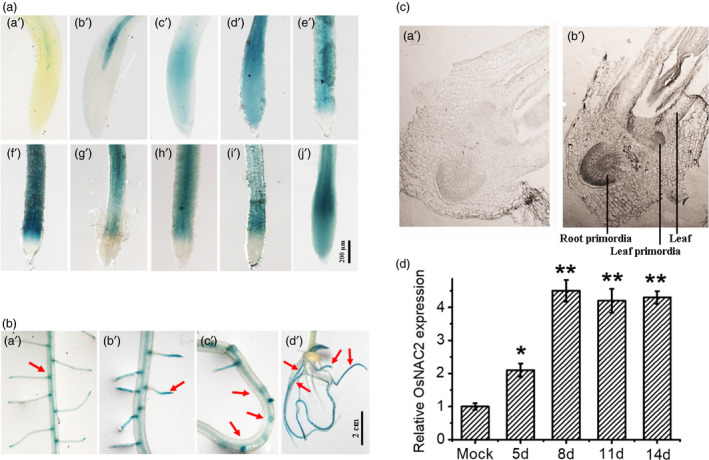
GUS staining in the root of transgenic rice plants expressing *OsNAC2* promoter: *GUS*. (a) The temporal distributions of GUS, a’–j’ means 4–14 days’ growth of rice roots. (b) The spatial distributions of GUS. (a’) the base of lateral root; (b’) the apex of lateral root; (c’) lateral root primordia; (d’) crown roots. (c) RNA in situ hybridization of *OsNAC2* in roots 5 days after germination with antisense (b’) and sense (a’) probes of *OsNAC2*. Scale bar = 10 μm. (d) *OsNAC2* expression in rice root after different days’ growth by qPCR. Relative mRNA level was calculated using the ΔΔC_T_ method from triplicate data. *OsActin* was used as internal control to normalize the different samples with the same amount of plant RNA. Seed roots of 4 days’ growth were set as 1× expression level.

To characterize the OsNAC2 function in root development, the spatial and temporal expression profiling of *OsNAC2* were investigated in transgenic plants expressing β‐glucuronidase (GUS) driven by the native promoter of *OsNAC2* promoter (1,947 bp). In 4‐ to 5‐day‐old transgenic seedlings, GUS activity was detected modestly in the intermediate section of the maturation zone (Figure 1a, a’ and b’). Two days later, GUS activity was observed in the whole root tip (Figure 1a, c’ and d’). In 9‐ to 13‐day‐old plants, GUS staining was detected mainly in the elongation zone (Figure 1a, e’ to I’). In 14‐day‐old plants, GUS activity spread to the whole root tip again (Figure 1a, j’). In addition, GUS staining was also detected in the apex and base of lateral roots, the lateral root primordia and crown roots (Figure 1b, a’ to d’). Consistent with these results, RNA *in situ* hybridization revealed that *OsNAC2* was highly expressed in the root primordia of 5‐day‐old seedlings (Figure 1c). Furthermore, *OsNAC2* was significantly induced during root early growth tested by qPCR (Figure 1d). Therefore, our results imply that *OsNAC2* might regulate root development.

### *OsNAC2* negatively regulates root growth

**Figure 2:**
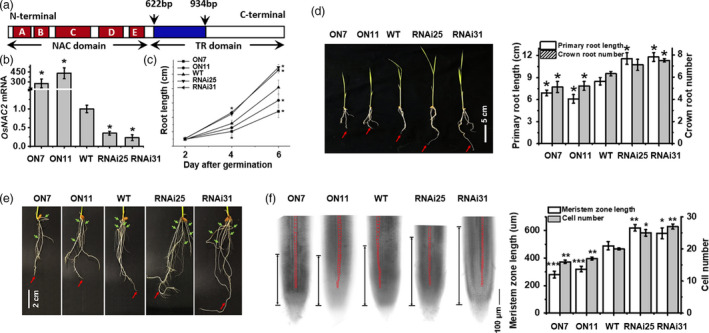
OsNAC2 regulates root length and meristem size. (a) Structure of the OsNAC2 and the arrows denoted the interfering fragment to construct RNAi vector. (a–e) represent five subdomains that are consensus in the N‐terminal region. (b) Expression of *OsNAC2* in different *OsNAC2*‐transgenic rice. (c) Root growth curves of WT and *OsNAC2* transgenic plants after germination. (d, e) 2‐week‐old primary (red arrows) and crown root (green arrows) phenotype of WT and *OsNAC2* transgenic plants. ON7 and ON11: *OsNAC2*‐overexpressing lines. RNAi25 and RNAi31: *OsNAC2‐* RNA interference lines. (e) showed the dispersed and magnified phenotype of crown root. (f) Bright‐field microscopy images of roots from 1‐week‐old *OsNAC2* transgenic and WT plants. The distance between the transition zone and the quiescent centre (two black arrows) corresponds to the meristem size. (g) Meristem size and the number of cortex cells in root meristems of 1‐week‐old transgenic and WT seedlings. Asterisks represent statistically significant differences between WT and transgenic plants. *P <0.05, **P < 0.01, ***P < 0.001, Student's *t‐*test.

To clarify the effects of OsNAC2 on root development, we further analysed previously described *OsNAC2‐*overexpressing lines (*OsNAC2‐OX*;* ON7* and *ON11*) and *OsNAC2* knockdown lines (*OsNAC2‐RNAi*;* RNAi25* and *RNAi31*) (Chen *et al*., 2015; Mao *et al*., 2017). Figure 2a shows the structure of the OsNAC2 coding sequence, and the arrows denoted the interfering fragment to construct RNAi vector. Moreover, we found that mRNA of *OsNAC2* was highly induced in overexpression lines, while inhibited in RNA interfering lines (Figure 2b). The primary roots were obviously shorter in *OsNAC2‐OX* lines while longer in *OsNAC2‐RNAi* lines, compared with the WT plants (Figure 2d). At the seedling stages, the *OsNAC2‐RNAi* plants produced more crown roots than the WT plants (Figure 2e). Additionally, the root growth rates of *ON7* and *ON11* plants were significantly lower than that of the WT plants, whereas the *OsNAC2‐RNAi* plants exhibited the opposite pattern (Figure 2c). Furthermore, we observed that the meristem zone length of *ON7* and *ON11* plants was shorter while that of *RNAi25* and *RNAi31* plants was longer than the WT plants (Figure 2f). We also demonstrated that the shorter meristem zone was consistent with the decreased number of cortical cells in a single row between the quiescent centre and the transition zone (Figure 2f). These results suggested that OsNAC2 negatively regulates the growth of meristem cells, thereby shortening root length.

### OsNAC2 increases cytokinin content and sensitivity in rice root

To study the molecular mechanisms underlying OsNAC2‐regulated root growth, we completed a microarray analysis to examine the differences in the global gene expression levels in *ON11* and WT roots. Compared with the WT, 343 up‐regulated and 363 down‐regulated genes were detected in the *ON11* roots (pfp < 0.05). Many of these genes were associated with cytokinin metabolism and signalling (e.g. *OsIPT*,* OsCKX* and *OsRR* genes; Table S2).

**Figure 3:**
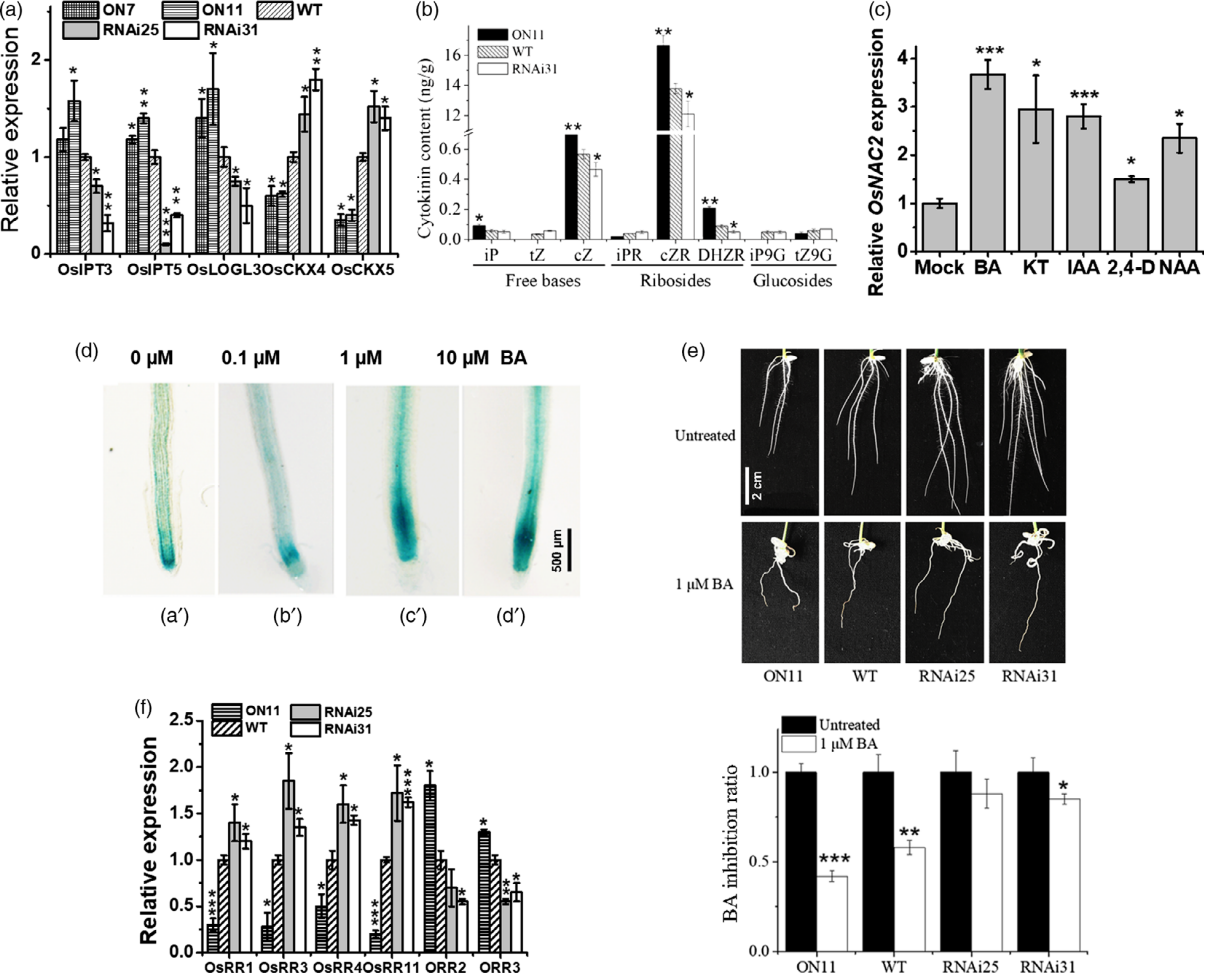
OsNAC2 altered cytokinin metabolism and sensitivity. (a) The expression of cytokinin biosynthesis‐ and degradation‐related genes in roots of 2‐week‐old WT and transgenic plants. (b) Quantification of endogenous cytokinin content in 2‐week‐old roots. iP, Isopentenyladenine; tZ, trans‐zeatin; cZ, cis‐zeatin. (c) Relative expression of *OsNAC2* in roots of 1‐week‐old WT in response to 10 μm BA, 10 μm KT, 10 μm IAA, 10 μm 2, 4‐D and 10 μm NAA. Data were means ± SE with five replicates. (d) GUS staining of *OsNAC2pro::GUS* seedlings under 0 μm (a’), 0.1 μm (b’), 1 μm (c’) and 10 μm (d’) BA treatment for 8 h. (e) Inhibition rate of 6‐day‐old WT and *OsNAC2* transgenic seedlings on the MS medium containing 0 or 1 μm BA. Data were means ± SE with at least 30 seedlings. (f) Relative expression of OsRRs and ORRs in roots of 2‐week‐old WT and *OsNAC2* transgenic seedlings. Data were means ± SE with five replicates.

We also examined the expression of cytokinin metabolism genes in a quantitative real‐time polymerase chain reaction (qPCR) assay. The mRNA level of cytokinin biosynthesis‐related genes (*OsIPT3*,* OsIPT5* and *OsLOGL3*) was significantly up‐regulated in *ON11*, which was consistent with the microarray results (Figure 3a). We also observed that the expressions of *OsCKX4* and *OsCKX5*, which encode cytokinin oxidase/dehydrogenase, were down‐regulated in *OsNAC2‐OX* lines, but up‐regulated in the *OsNAC2‐RNAi* lines (Figure 3a). Cytokinins reportedly inhibit rice root formation (Durbak *et al*., 2012). To determine whether the transgenic phenotypes were caused by cytokinin accumulation, the endogenous cytokinin content was examined by tandem mass spectrometry. The abundance of cytokinin free bases (iP and cZ) and ribosides (cZR and DHZR) was significantly higher in *ON11*, but lower in *RNAi31*, compared with the WT (Figure 3b). These results implied that *OsNAC2* stimulates cytokinin accumulation by repressing cytokinin degradation and promoting cytokinin biosynthesis.

Since cytokinin and auxin are the two main plant hormones regulating root development, we checked whether *OsNAC2* is induced by these hormones. We treated 1‐week‐old WT seedlings with 6‐benzylaminopurine (BA), 6‐furfurylaminopurine (kinetin, KT), IAA, naphthaleneacetic acid (NAA) or (2, 4‐dichlorophenoxy)‐acetic acid (2, 4‐D) for 8 h. qPCR revealed that *OsNAC2* was highly induced by BA, KT, IAA and NAA, and slightly induced by 2, 4‐D (Figure 3c). In addition, GUS staining was obviously stronger at the root tip when treated with 1 and 10 μm exogenous BA, compared with the normal condition (Figure 3d).

To further assess the sensitivity of the transgenic lines to cytokinin, rice seeds were germinated on Murashige and Skoog (MS) medium with or without 1 μm BA. As expected, cytokinin showed stronger inhibition on root growth of *ON11* plants than the WT, while weaker on that of *RNAi* plants (Figure 3e), which indicated that OsNAC2 mediates cytokinin‐dependent root growth.

To clarify whether cytokinin signalling is regulated by OsNAC2, the expressions of cytokinin type‐A and type‐B RR genes were analysed by qPCR. In *OsNAC2‐RNAi* plants, expressions of the four tested type‐A RR genes (*OsRR1*,* OsRR3*,* OsRR4* and *OsRR11*) were significantly up‐regulated, while that of type‐B RR genes (*ORR2* and *ORR3*) were repressed (Figure 3f). Given that type‐A RRs negatively regulate cytokinin responses, while type‐B RRs positively regulate cytokinin signalling (To and Kieber, 2008), our results suggested that OsNAC2 positively influences cytokinin signalling.

### *OsNAC2* alters the expression of genes related to the IAA metabolic pathway

**Figure 4:**
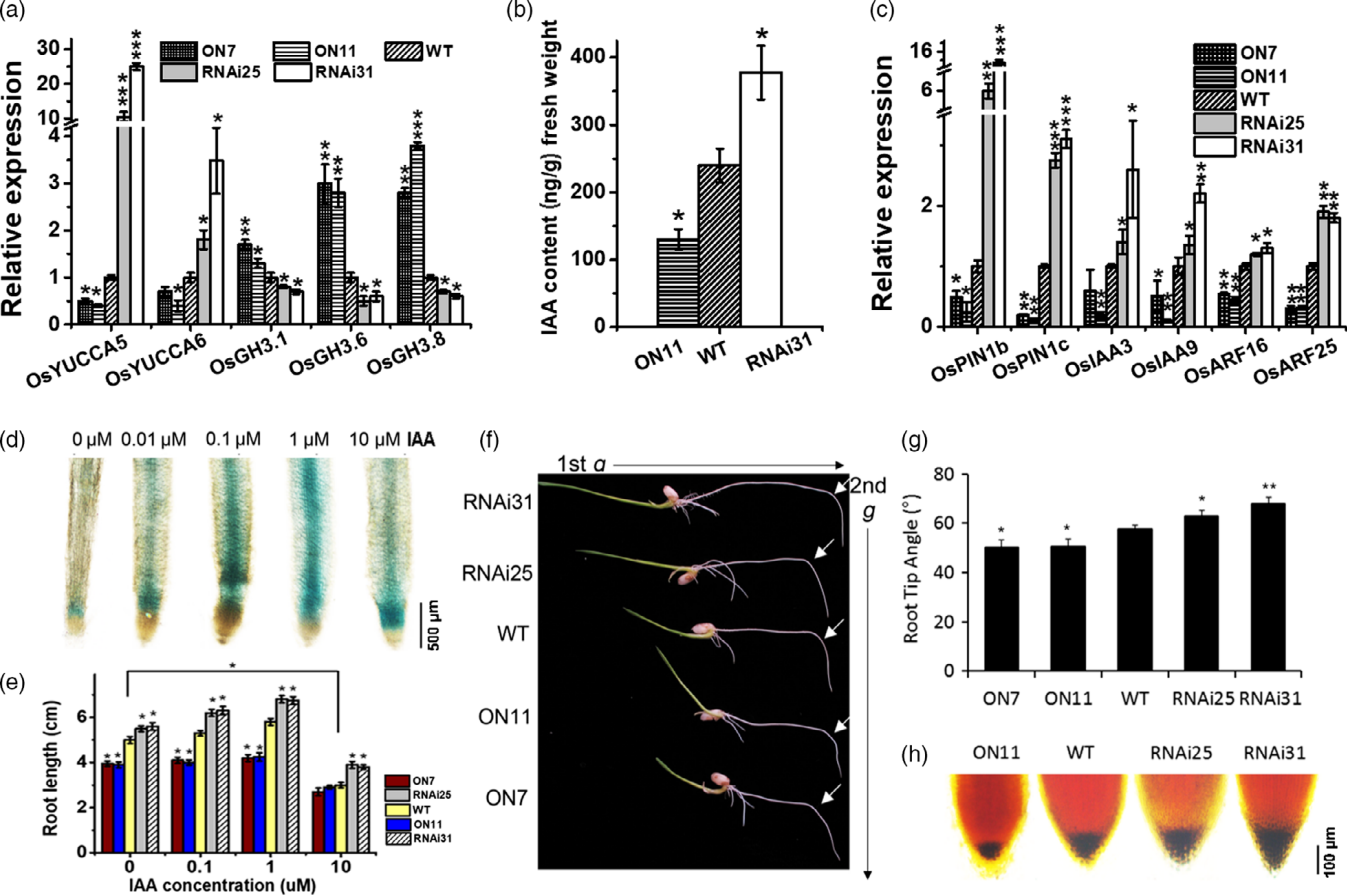
Auxin metabolism and responses in *OsNAC2* transgenic rice. (a) Expression of auxin biosynthesis‐related genes in the roots of *OsNAC2* transgenic seedlings and the WT. (b) Quantification of free IAA content in 2‐week‐old roots. (c) Expression of auxin response‐related genes in roots of 2‐week‐old WT and *OsNAC2* transgenic seedlings. (d) GUS staining of *OsNAC2pro::GUS* seedlings under 0, 0.01, 0.1, 1, 10 μm IAA treatment for 8 h. (e) IAA‐treated WT and *OsNAC2* transgenic seedlings at 6 DAG cultured in MS medium with or without 0.1 μm IAA. (f, g) Comparison of gravitropic sensitivity of root tip between *OsNAC2* transgenic seedlings and the WT. Data were means ± SE with at least 15 seedlings. (h) KI/I_2_ staining of seedling root tips. Amyloplasts are shown in dark brown. Data were means ± SE with five replicates.

Indole‐3‐acetic acid, an auxin phytohormone, is essential for root development because of its effects on root meristem cell division, lateral root formation, emergence and root elongation (Saini *et al*., 2013). In the current study, a microarray analysis revealed that the expressions of genes related to IAA metabolism and signalling were obviously altered in *ON11* plants (Table S3). Moreover, qPCR assay confirmed that in *OsNAC2‐RNAi* plants, IAA biosynthesis‐related genes (*OsYUCCA5* and *OsYUCCA6*) were significantly up‐regulated, while those of IAA conjugation/inactivation‐related genes (*OsGH3.1*,* OsGH3.6* and *OsGH3.8*) were down‐regulated (Figure 4a). These results indicated that OsNAC2 decreases the abundance of the biologically active form of IAA (free IAA). The tandem mass spectrometry analysis confirmed that the endogenous free IAA level was significantly higher in *RNAi31* (378 ng/g fresh weight) than in WT (240 ng/g fresh weight) (Figure 4b). Thus, it appears that OsNAC2 decreases IAA contents by down‐regulating the expressions of IAA biosynthesis‐related genes and up‐regulating that of IAA inactivation‐related genes.

### Knockdown of *OsNAC2* enhances the auxin responses of rice roots

*OsNAC2pro::GUS* transgenic plants were used to confirm the *OsNAC2* expression pattern. As the growth of exogenous IAA, GUS activity increased at the root tip (Figure 4d). Furthermore, in the presence of 0.1 and 1 μm IAA, the roots of *OsNAC2‐RNAi* plants were significantly longer than the untreated roots, whereas the *OsNAC2‐OX* lines did not show obviously changes (Figure 4e). Interestingly, all the five transgenic lines were obviously inhibited by 10 μm IAA, which indicated that OsNAC2 modulated root development in IAA dose‐dependent manner. To further investigate whether IAA response altered by OsNAC2, we checked the expressions of genes contributing to auxin transport and signalling. The result showed that mRNA levels of auxin transport‐related genes (*OsPIN1b* and *OsPIN1c*), transcriptional regulators (*OsARF25*) and IAA family members (*OsIAA3*,* OsIAA9* and *OsIAA16*) were all up‐regulated in the *OsNAC2‐RNAi* lines, while down‐regulated in *OsNAC2‐OX* lines (Figure 4c; Table S3).

The root gravitropic response has been reported to be regulated by differential distribution of auxin (Ottenschläger *et al*., 2003). To gain a further understanding of the relationship between OsNAC2 and auxin response, we examined gravity‐induced root curvature of *OsNAC2*‐transgenic plants after gravistimulation at 90° to the vertical for 24 h. The average root tip angles in *RNAi25* and *RNAi31* plants were 63° and 68°, respectively, which were significantly larger than that in the WT plants (57°), whereas that in *ON7* and *ON11* plants were 50° and 51°, respectively (Figure 4f,g). A previous study proved the accumulation of amyloplasts showed a linear relationship to the gravistimulation (Sack *et al*., 1984; Sack, 1997). Thus, we also examined amyloplast accumulation in *OsNAC2*‐transgenic plants and found that *OsNAC2‐RNAi* plants had more while *OsNAC2‐OX* plants had fewer amyloplasts, compared with the WT root tips (Figure 4h). Taken together, the impaired root gravitropism suggested the role OsNAC2 played in the auxin responses of rice roots.

### OsNAC2 binds directly to promoters of cytokinin‐ and auxin‐related genes

**Figure 5:**
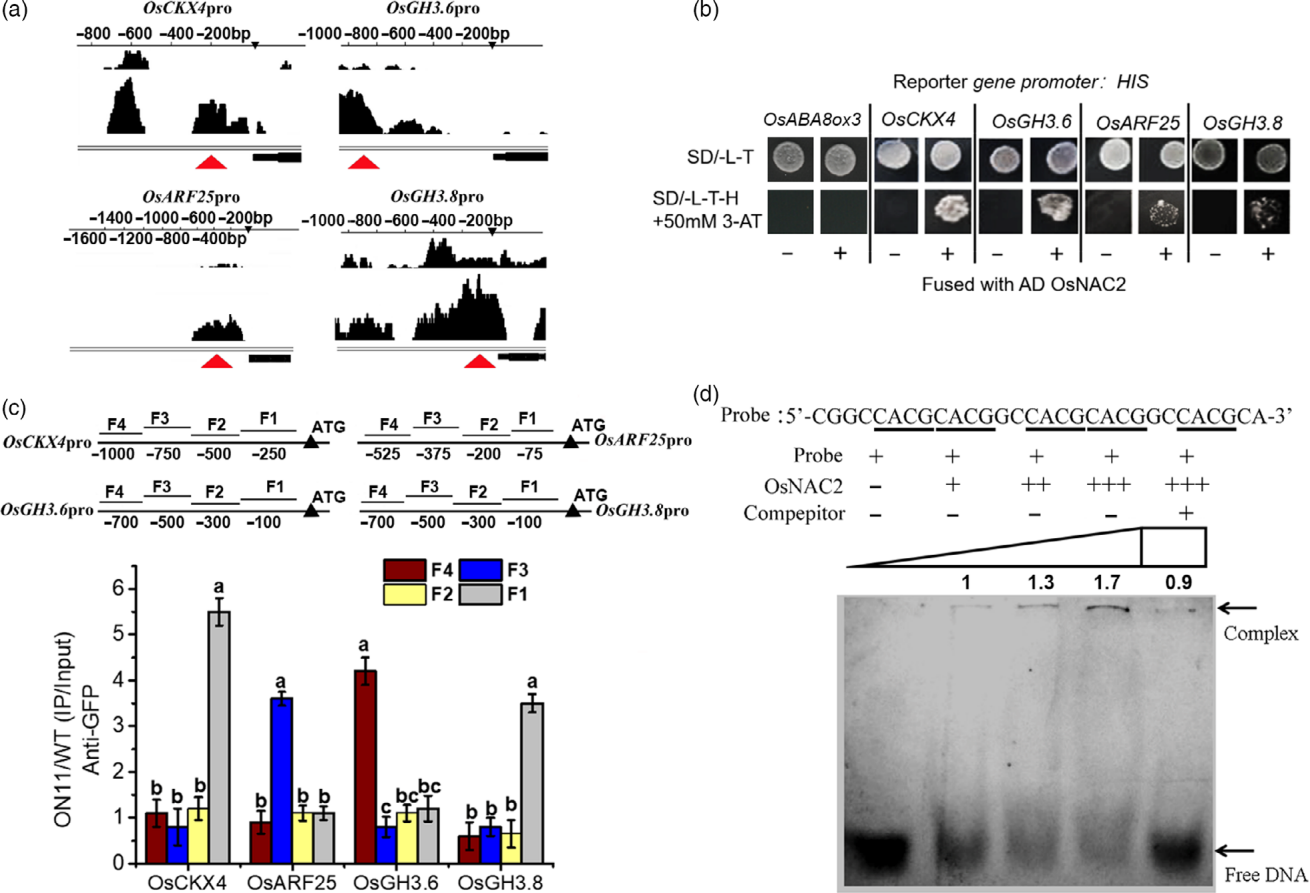
OsNAC2 directly regulates expression of *OsCKX4, OsARF25, OsGH3.6* and *OsGH3.8*. (a) Binding peaks of *OsCKX4, OsARF25, OsGH3.6* and *OsGH3.8* in ChIP‐seq assay. Black peaks represent for sequence hits on DNA of each gene region. The higher the peak, the more the binding in this region. The bars above the peak show the distance from the ATG start codon of each gene. The black bar under the peak represents for the coding area of each gene. Red arrows show the possible enriched reign of the promoters immunoprecipitated by anti‐GFP antibody. (b) Yeast one‐hybrid assays of the interaction between OsNAC2 and the promoter of *OsCKX4, OsARF25, OsGH3.6* and *OsGH3.8*. *OsABA8ox3* is used as a negative control that is not regulated by OsNAC2. (c) ChIP‐qPCR assays. Total protein extracted from *35S::OsNAC2‐mGFP* transgenic plants grown for 2 weeks was immunoprecipitated with an anti‐GFP antibody. Fragmented genomic DNA was eluted from the protein–DNA complexes and subjected to qPCR analysis. The long black bars represent for promoter regions which were amplified. The numbers under the bar show the distance from the ATG start codon. Short bars represent for the corresponding region of each pair of primers on the promoter. (d) EMSA with recombinant OsNAC2 protein and FAM‐labelled oligonucleotide (5′‐CGGCCACGCACGGCCACGCACGGCCACGCA‐3′, designed based on the enriched fragment of OsCKX4 promoter by ChIP‐qPCR), and a competitive assay using the unlabelled probe of this oligonucleotide. The binding experiments were performed using increasing levels of OsNAC2 protein. The intensity of the shifted fragments was accurately quantified by the ImageJ2x program.

To investigate whether OsNAC2 directly targets the genes in auxin and cytokinin metabolism and signalling pathways, a ChIP‐seq experiment was conducted to scan the promoter region of the genes of interest. We detected binding peaks for four of the candidate genes (*OsCKX4*,* OsARF25*,* OsGH3.6* and *OsGH3.8*) (Figure 5a) in *OsNAC2‐*overexpression line *ON11*, compared with the WT. Second, yeast one‐hybrid was performed to test direct interaction between OsNAC2 and DNA. Our result showed that the GAL4 transcriptional activation domain fused with OsNAC2 activates the HIS3 reporter gene driven by the promoter of *OsCKX4*,* OsARF25*,* OsGH3.6* and *OsGH3.8* (Figure 5b). Third, ChIP‐qPCR assay involving anti‐green fluorescent protein (GFP) antibodies showed that specific fragments in the promoters of *OsCKX4*,* OsARF25*,* OsGH3.6* and *OsGH3.8* were significantly enriched in the anti‐GFP antibody‐immunoprecipitated DNA, compared with the negative control in which DNA amplified from WT plants (Figure 5c). These data suggested that OsNAC2 regulates IAA and cytokinin functions by specifically binding to the promoters of *OsCKX4*,* OsARF25*,* OsGH3.6* and *OsGH3.8*.

The DNA‐binding motif of the NAC domain comprises a 4‐bp core sequence, CACG (Olsen *et al*., 2005). We previously determined that CACG sequences exist in the promoters of OsNAC2‐targeted genes (Mao *et al*., 2017). The ChIP‐qPCR assay in the current study confirmed that CACG is present in the *OsCKX4*,* OsARF25*,* OsGH3.6* and *OsGH3.8* promoters (Figure S1). An electrophoretic mobility shift assay (EMSA) was conducted with a probe that included a 10‐bp sequence (CGGCCACGCA) in triplicate. The probe was designed based on the enriched fragment of *OsCKX4* promoter by ChIP‐qPCR. We observed that the mobility of this fragment was obviously shifted in the presence of OsNAC2 protein (Figure 5d). In a competition experiment, the ability of OsNAC2 to bind with CACG was considerably weakened by an unlabelled probe (Figure 5d). Consequently, the CACG fragment is the possible binding motif for OsNAC2.

It has been reported that cytokinin‐responsive type‐A OsRRs also reportedly bind directly to *OsCKX4* promoter (Gao *et al*., 2014). Thus, we hypothesize that OsNAC2 could be in cooperation with OsRRs to modulate *OsCKX4* expression in rice root growth. To determine whether OsNAC2 interacts with OsRRs, we performed Y2H assays using OsRRs as the bait and OsNAC2 as the prey. We found that neither OsRR4 nor OsRR6 interacts with OsNAC2 on SD/‐His/‐Trp/‐Leu/‐Ade plate. Furthermore, due to the self‐activation of OsRR10, Y2H was performed using N‐terminal domain of OsNAC2 as the bait and OsRRs as the prey. There still appears to be a nonspecific interaction between OsNAC2 and OsRR4, OsRR6, or OsRR10 (Figure S2).

### *OsNAC2* negatively regulates root formation *via* depressing *OsCDK* and *OsCRL* pathways

It has been reported that cytokinin inhibits root elongation by decreasing root cell division (Werner *et al*., 2003). In the previous study, the *OsNAC2‐OX* plants had a smaller root meristem zone with fewer cells than the WT plants. Thus, we investigated the expression of cell cycle marker genes, cyclin‐dependent protein kinase (CDK) (Endo *et al*., 2012), in a microarray assay. Our data suggested that the mRNA levels of most of these genes were down‐regulated in the *OsNAC2‐OX* line (Table S4). Our qPCR assay confirmed that the *cdc2Os‐1*,* cdc2Os‐2* and *CDKB1;1* mRNA levels were up‐regulated in the *OsNAC2‐RNAi* lines, but down‐regulated in the *OsNAC2‐OX* lines (Figure S3).

The *OsCRL* genes, which function downstream of auxin response factors (ARFs), are involved in different regulatory pathways of crown root initiation and growth (Coudert *et al*., 2015; Kitomi *et al*., 2011a; Liu *et al*., 2009). The qPCR data indicated that expressions of *OsCRL1*,* OsCRL4* and *OsCRL5* were obviously up‐regulated in *OsNAC2‐RNAi* lines, but were down‐regulated in *OsNAC2‐OX* lines (Figure S3). Therefore, OsNAC2 appears to negatively regulate elongation of primary and crown roots, *via* depressing *OsCDK* and *OsCRL* pathways.

### *OsGH3.6* and *OsCKX4* are downstream targets of OsNAC2 according to a genetic analysis

**Figure 6:**
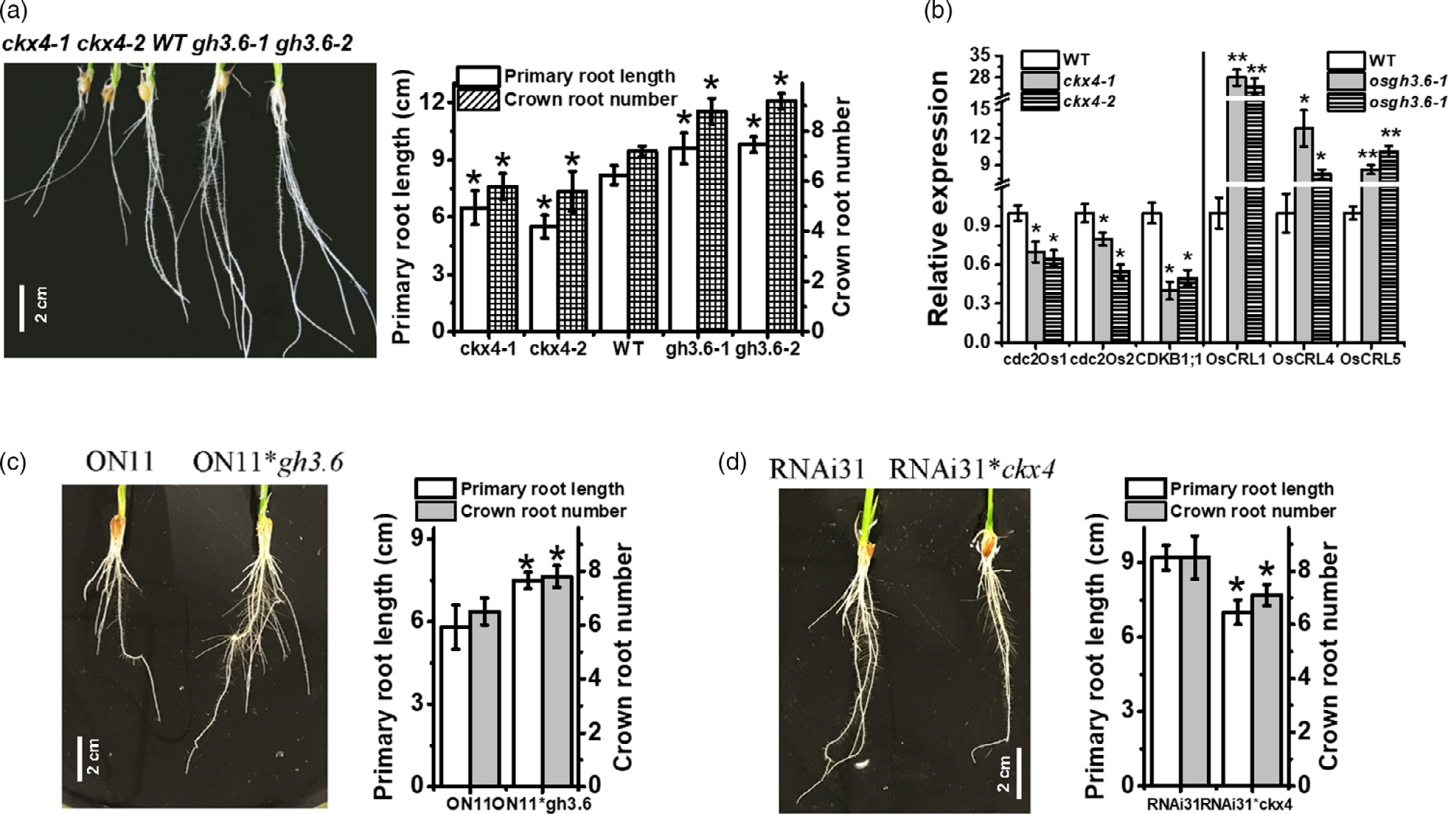
Genetic analysis of *ckx4* and *gh3.6* mutants and hybrid homozygote. (a) Root phenotype of 2‐week‐old WT,* ckx4* and *gh3.6* plants. Data were means ± SE with 10 replicates. (b) The expression of *OsCRL* and *OsCDK* genes in 2‐week‐old *ckx4* and *gh3.6* mutants. (c, d) Root phenotype of 2‐week‐old ON11/ *osgh3.6* and RNAi31/ *osckx4* homozygote.

To test whether *OsGH3.6* and *OsCKX4* help mediate root formation, we analysed the *gh3.6* (*gh3.6‐1* and *‐2*) and *ckx4* (*ckx4‐1* and *‐2*) tilling mutants (Figures S4 and S5). Compared with the WT plants, the *gh3.6‐1* and *gh3.6‐2* plants had longer primary root and more crown roots, similar to that of *OsNAC2‐RNAi* plants. Meanwhile, the *ckx4‐1* and *ckx1‐2* plants had shorter primary roots and less crown roots, similar to that of *OsNAC2‐OX* plants (Figure 6a). Consistent with these observations, *OsCDK* expressions were down‐regulated in *ckx4* roots, while that of *OsCRL* were up‐regulated in *gh3.6* roots (Figure 6b). These data indicated that *OsGH3.6* and *OsCKX4* are involved in OsNAC2‐regulated root initiation and elongation.

To further test whether OsNAC2 regulates root formation *via* its effects on *OsGH3.6* and *OsCKX4*, we crossed *osgh3.6* or *osckx4* plants with *ON11* or RNAi31 plants, and the resulting hybrid homozygote was analysed. The identification of each hybrid homozygote is presented in Figure S6. The primary root of the ON11/*osgh3.6* hybrid homozygote was longer than that of *ON11* plants, similar to that of *osgh3.6* plants (Figure 6c). In contrast, the *RNAi31*/*osckx4* hybrid homozygote produced a relatively short primary root, similar to that of *osckx4* plants (Figure 6d). Overall, these data strongly suggested that *OsGH3.6* and *OsCKX4* functioned downstream of OsNAC2.

### Targeted mutagenesis of *OsNAC2* using the CRISPR/Cas9 system alters rice root development

**Figure 7:**
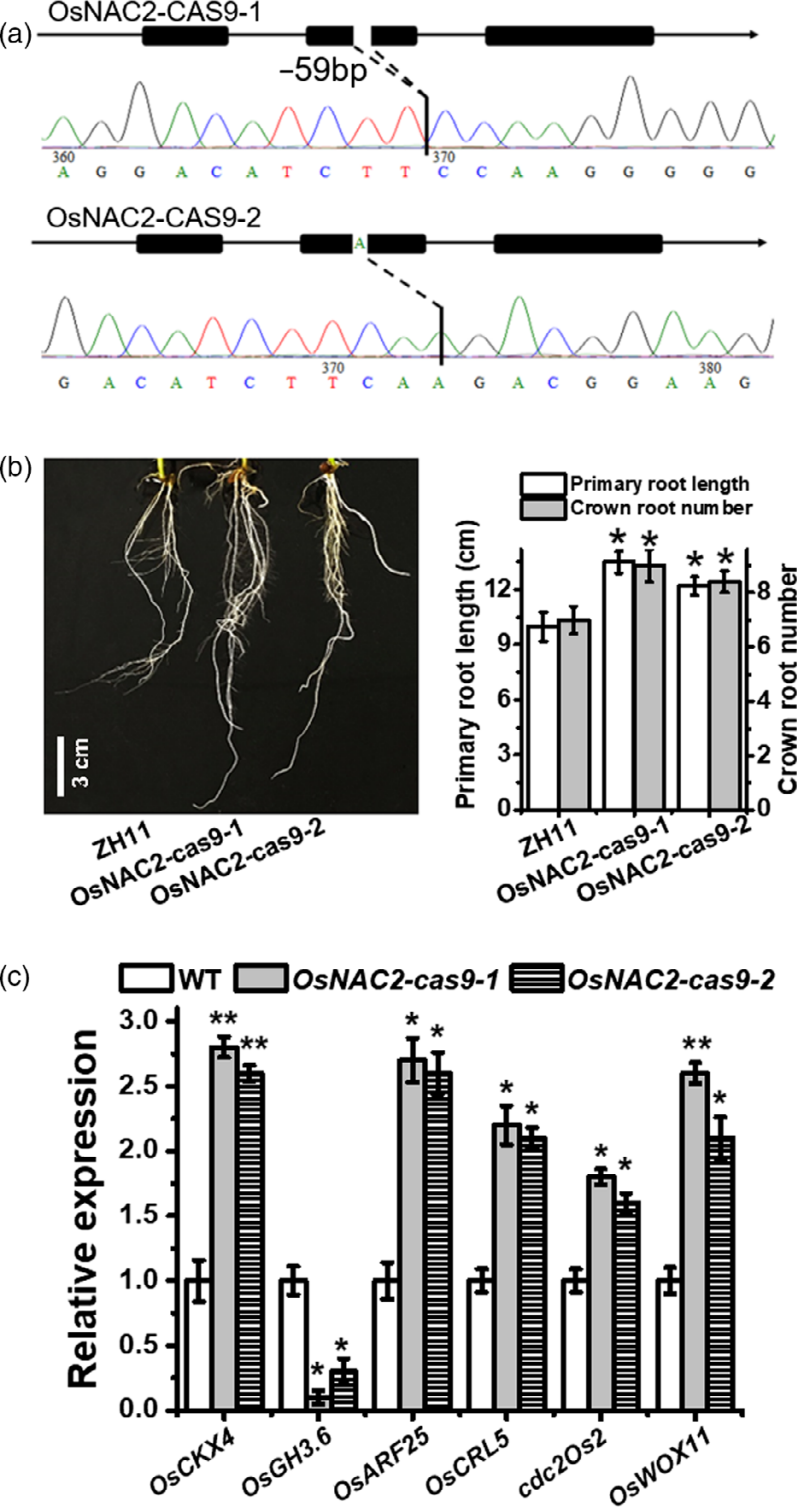
Verification and phenotype and of OsNAC2‐knockout lines. (a) Verification of OsNAC2‐knockout lines by PCR‐based sequencing. Two representative transgenic lines (abbreviated as OsNAC2‐cas9‐1 and OsNAC2‐cas9‐2, respectively) for OsNAC2 knockout are generated from ZH11 genetic background. (b) Root phenotype of 2‐week‐old ZH11 and OsNAC2‐knockout plants. Data were means ± SE with 10 replicates. c, The expression of *OsCKX4*,* OsGH3.6*,* OsARF25*,* OsCRL5*,* cdc2Os2* and *OsWOX11* in 2‐week‐old ZH11 and OsNAC2‐knockout plants.

To further investigate the role of OsNAC2 in root development, the CRISPR/Cas9 system was applied to knock out the endogenous *OsNAC2* gene in a WT line (ZH11). Lines *OsNAC2‐cas9‐1* and *OsNAC2‐cas9‐2*, with a 59‐ and 1‐base deletion in the target site, respectively, were evaluated for further analysis (Figure 7a). As expected, the roots of *OsNAC2‐cas9* plants were longer primary roots and more crown roots than the ZH11 roots (Figure 7b). Additionally, the mRNA levels of IAA signalling‐related and cytokinin oxidase genes were up‐regulated, whereas that of IAA inactivation‐related genes were down‐regulated (Figure 7c), which was essentially consistent with the analysis of *OsNAC2‐RNAi* plants.

### OsNAC2 indirectly mediates *OsWOX11* expression

A previous study concluded that OsWOX11 promotes cell division in the root meristem and integrates auxin and cytokinin signals to stimulate crown root development (Zhao *et al*., 2015). Thus, we examined whether OsNAC2 regulates root development through OsWOX11. Our qPCR results showed that *OsWOX11* expression was obviously repressed in *OsNAC2‐OX* lines, but was induced in *OsNAC2‐RNAi* and *OsNAC2‐cas9* lines (Figure 7c and Figure S7a). Additionally, *OsNAC2* expression was up‐regulated in the *oswox11* mutant (Figure S7a). These results suggested there may be an antagonistic relationship between OsNAC2 and OsWOX11. Furthermore, yeast one‐hybrid and yeast two‐hybrid assays were performed to validate the interactions among OsNAC2, OsWOX11 and their promoters. We observed that OsNAC2 does not interact with OsWOX11, nor does it directly target the *OsWOX11* promoter (Figure S7b). Thus, it seems that OsNAC2 indirectly mediates the expression of *OsWOX11*.

## Discussion

### OsNAC2 affects root growth via regulating *OsCDK* and *OsCRL* expressions

The root system is required for water and nutrient uptake, anchoring and storage (Coudert *et al*., 2010), and optimizing root system architecture can overcome yield limitations in crop plants due to water or nutrient shortages (Werner *et al*., 2010). The plant‐specific NAC transcription factor genes have multiple roles related to plant growth and development. It has been reported that OsNAC5, OsNAC9 and OsNAC10 are associated with significantly thick roots and enhanced drought tolerance at the reproductive stage (Jeong *et al*., 2010, 2013; Redillas *et al*., 2012). In this study, we identified and characterized OsNAC2 negatively regulates root growth and crown root number (Figure 2d,e). Moreover, diverse root meristem sizes in different *OsNAC2* transgenic plants suggest that OsNAC2 represses cell division in root meristems (Figure 2f). Our qPCR results revealed that the mRNA levels of cell cycle marker genes (e.g. *cdc2Os‐1*,* CDKB2;1* and *CDKB1;1*) and crown root initiation‐related genes (e.g. *OsCRL4* and *OsCRL5*) were down‐regulated in *OsNAC2‐OX* lines (Figure S3, Tables S3 and S4). Consequently, we speculated that OsNAC2 inhibits root initiation and elongation, probably by down‐regulating the expression of *OsCDK* and *OsCRL* genes.

### OsNAC2 integrates upstream auxin and cytokinin metabolic and signalling to control root development

Auxin acts as a versatile trigger in root developmental processes, including cell elongation, cell division, gravitropism, root initiation and apical dominance (Benková *et al*., 2009). It has been reported that auxin enhances the transcription of several classes of early genes, such as the Aux/IAA, GH3 and SMALL AUXIN UP RNA (SAUR) gene family members (Abel and Theologis, 1996). Among them, GH3 proteins are responsible for converting active IAA to its inactive form via the conjugation of IAA with amino acids (Staswick *et al*., 2005). Meanwhile, auxin response factors (ARFs) are transcription factors that bind in promoters of primary/early auxin response genes (Wang *et al*., 2007). Nowadays, crosstalk between auxin and cytokinin was confirmed to be required for the coordinated expression of a series of genes in rice roots. For example, down‐regulation of OsARF1‐targeted *OsCRL5* gene could activate *OsRR1* expression and affect cytokinin signalling (Kitomi *et al*., 2011a). *OsCKX4*, which encodes a cytokinin oxidase/dehydrogenase, is directly regulated by OsARF25 and OsRRs, and stimulates crown root formation (Gao *et al*., 2014). However, the upstream network of the auxin and cytokinin signalling pathways in root development remains unclear. In this study, we observed that the IAA content was significantly lower and the cytokinin content was higher in *OsNAC2‐OX* transgenic plants than in WT plants (Figures 3b and 4b). qPCR data suggested that in *OsNAC2‐OX* plants, biosynthesis and responses of auxin were weakened, while that of cytokinin were enhanced (Figures 3 and 4). Further analyses revealed that OsNAC2 could bind directly to the promoters of IAA inactivation‐related genes (*GH3.6* and *GH3.8*), an IAA response gene (*OsARF25*) and a cytokinin oxidase gene (*OsCKX4*) (Figure 5). A genetic analysis indicated that ON11/ *osgh3.6* hybrid homozygote produced a relatively strong root system, similar to that of *osgh3.6* plants, while *RNAi31*/*osckx4* hybrid homozygote produced a relatively weak root system, similar to that of *osckx4* plants (Figure 6). These data strongly suggested that *GH3.6*,* GH3.8*,* OsARF25* and *OsCKX4* functioned downstream of OsNAC2, consistent with the conclusion that OsNAC2 retard root growth via the integration of auxin and cytokinin pathways.

### The OsNAC2 regulatory network controls root development indirectly through the WOX pathways

The CRL1/ARL1‐CRL5 pathways affect crown root initiation by auxin (Kitomi *et al*., 2011a). Zhao *et al*. found that ERF3 and WOX11 pathways regulating crown root development differ from the CRL1‐CRL5 pathway, since CRL5 expression was unlikely to be regulated directly by ERF3 (Zhao *et al*., 2015). In the current study, *OsWOX11* expression was affected in different OsNAC2 transgenic plants, and OsNAC2 did not interact with OsWOX11 or bind to the *OsWOX11* promoter (Figure 7c and Figure S7). Interestingly, *OsNAC2* expression was also up‐regulated in the *oswox11* mutant, and OsWOX11 did not target the *OsNAC2* promoter (Figure S7). Thus, OsNAC2 and OsWOX11 pathways may be connected by a regulator that mediates their antagonistic relationship. Additional analysis of protein interactions is needed to explore the regulator of the relationship between the OsNAC2 and OsWOX11 pathways, which may clarify the molecular regulatory mechanism underlying rice root formation.

### OsNAC2 differentially regulates type‐A and type‐B RRs in rice

The RR proteins are part of the two‐component sensor‐regulator system related to cytokinin signal perception and transduction (Jain *et al*., 2006c). Type‐A RRs are defined as cytokinin primary RRs and negatively modulate cytokinin signalling (D'Agostino *et al*., 2000). The overexpression of *OsRR2*,* OsRR3* or *OsRR5* enhances the root system phenotype (Cheng *et al*., 2010; Zhao *et al*., 2015). In *A. thaliana*, the root meristems of the *arr3*,* 4*,* 5*,* 6*,* 7*,* 8*,* 9* and *15* mutants are obviously smaller than that of the WT plants (Zhang *et al*., 2011). Type‐B RRs are cytokinin signalling effectors, and most of them positively regulate cytokinin responses (To and Kieber, 2008). Mutations to *ARR1*,* ARR2*,* ARR10‐12* and *ARR18* may relieve the cytokinin‐induced inhibition of seedling root elongation (Mason *et al*., 2005; Sakai and Oka, 2001; Yokoyama *et al*., 2007). Collectively, type‐A RRs inhibit cytokinin signalling, positively regulate root meristem size, and promote root initiation and elongation, whereas type‐B RRs exhibit the opposite effects in developing roots. In this study, we observed that in *OsNAC2‐OX* plants, the mRNA levels of type‐A RR genes (*OsRR1*,* OsRR3*,* OsRR4* and *OsRR11*) were obviously down‐regulated, while that of type‐B RR genes (*ORR2* and *ORR3*) were up‐regulated (Figure 3f, Table S2). These findings suggest that OsNAC2 positively regulates cytokinin signalling, weakens root meristem activity and finally inhibits root formation.

### OsNAC2 contributes to cytokinin activities that restrict cell division

Cytokinins are a class of phytohormones that are important for plant growth and development. Previous studies documented that cytokinin inhibits root elongation by decreasing root meristem size (Beemster and Baskin, 2000; Dello Ioio *et al*., 2007; Werner *et al*., 2003). For example, the cytokinin biosynthesis triple mutant *ipt3,ipt5,ipt7* has abnormally low cytokinin contents and relatively large root meristems, resulting in an enhanced root growth rate and longer primary roots (Dello Ioio *et al*., 2007). Similarly, *AtCKX‐*overexpressing plants produce longer roots and have a larger root apical meristem with more cells compared with WT plants (Werner *et al*., 2003). Furthermore, the stronger root system in *AtCKX1*‐overexpressing lines was mainly attributed to the increased number of dividing cells in root meristems (Werner *et al*., 2003), which indicates that cytokinins regulate the root growth rate by controlling cell division, ultimately affecting the size of root apical meristems. In this study, we observed that OsNAC2 down‐regulates the expression of *OsCKX4* and type‐A RR genes (Figures 3 and 7). Additionally, root meristem sizes and cell numbers decreased considerably in *OsNAC2‐OX* lines (Figure 2f). The mRNA levels of the cell cycle marker genes were also obviously down‐regulated by OsNAC2 (Figure S3). We propose that OsNAC2 decreases cytokinin contents and signalling, inhibits cell division and finally restricts root elongation.

### OsNAC2 working model and its potential application in agriculture

**Figure 8:**
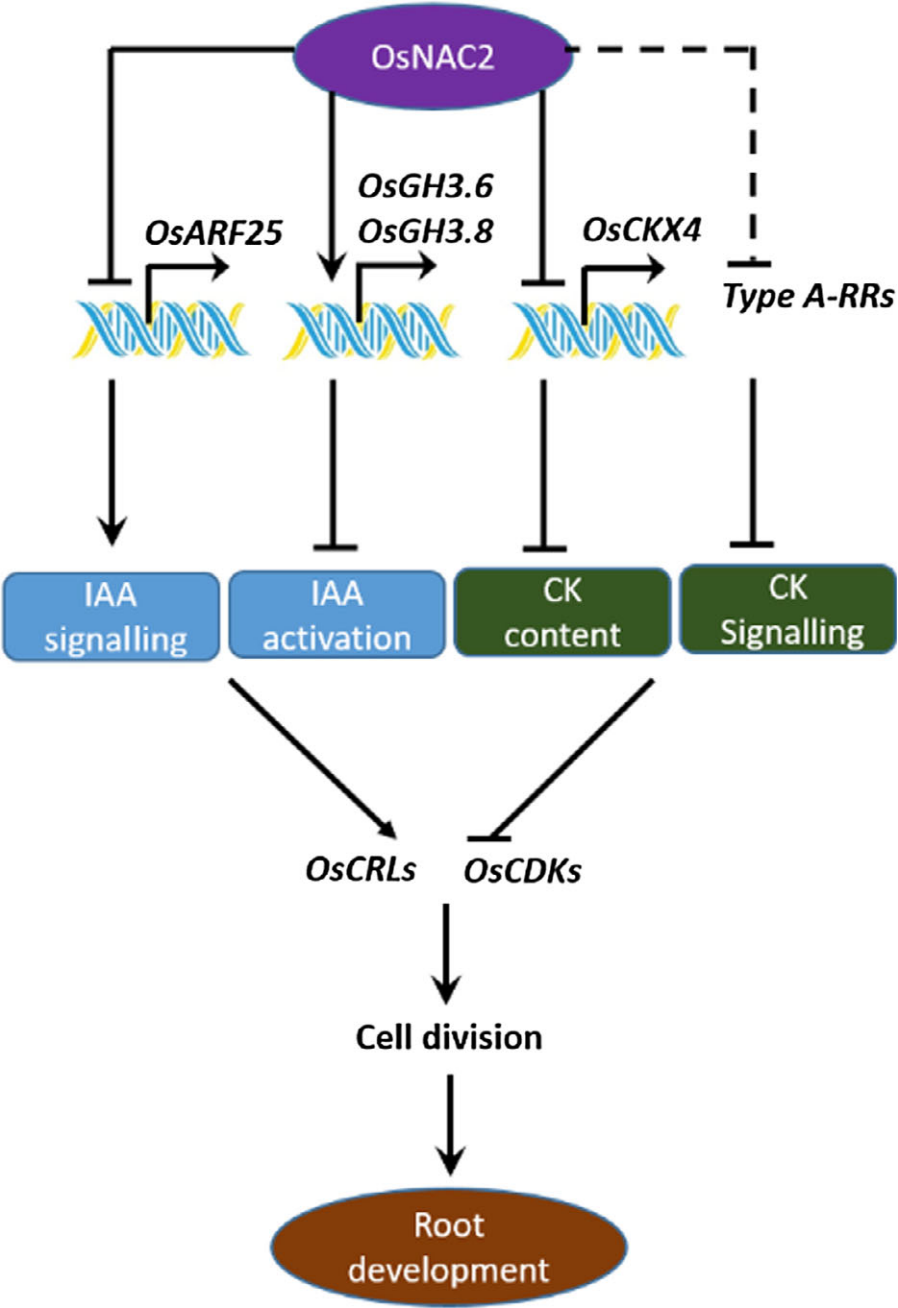
Proposed model for OsNAC2 action in root development of rice.

Based on the above results, we developed a working model for OsNAC2 functions related to rice root formation. Specifically, OsNAC2 directly regulates the expression of *GH3.6*,* GH3.8*,* OsARF25* and *OsCKX4*, which subsequently enhances cytokinin responses, but weakens auxin responses. This crosstalk between cytokinin and auxin pathways down‐regulates the expression of *OsCDK* and *OsCRL* genes, which inhibits root initiation and elongation *via* decreased cell division (Figure 8). In conclusion, our study revealed a regulatory network that functions upstream of auxin and cytokinin during rice root development.

Plants with stronger root systems increase the ability to absorb nutrients and water, and survive under abiotic stress or nutrient‐deficient conditions, supporting higher yield (Hodge *et al*., 1999; Liao *et al*., 2001). We demonstrated here that the rice transcription factor OsNAC2 is a potentially valuable candidate for genetic engineering of high‐yielding crops. Using RNA interfering technology, the primary roots were obviously longer and more crown roots in *OsNAC2‐RNAi* plants compared with the WT plants (Figure 2d,e). By CRISPR/Cas9 system, *OsNAC2* knockout showed stronger root system than ZH11 (Figure 7b). We previously reported that reduced *OsNAC2* expression leads to about 10% increase in the grain yield of RNAi lines (Mao *et al*., 2017). Furthermore, OsNAC2 directly regulates *GH3.6* and *OsCKX4* expressions in auxin and cytokinin pathways (Figure 8). By tilling technology, *gh3.6‐1* and *gh3.6‐2* plants had enlarged root system, similar to that of *OsNAC2‐RNAi* plants (Figure 6). The *RCc3pro*::*OsCKX4* transgenic plants were also found to exhibit enhanced root phenotypes (Gao *et al*., 2014). Thus, the data presented herein may be a useful strategy for manipulating roots to improve cereal crop yields *via* the auxin‐OsNAC2‐cytokinin pathway.

### OsNAC2 targets different downstream genes to regulate multiple plant functions

In the previous and current study, we demonstrate that OsNAC2 plays significant roles in multiple biological functions. OsNAC2 binds to the CACG fragment sequences in their respective target genes’ promotors performing different physiological functions. First, OsNAC2 directly regulates the expression of *GH3.6*,* GH3.8*,* OsARF25* and *OsCKX4*, which subsequently enhances cytokinin responses, but weakens auxin responses in root growth. Second, OsNAC2 interacts with the promoters of *OsEATB* and *OsKO2*, key genes of the GA pathway, to negatively regulate plant height and flowering time (Chen *et al*., 2015). Third, endogenous abscisic acid is accumulated in rice leaves by OsNAC2 targeting the promotors of ABA synthesis‐related genes (Mao *et al*., 2017). Fourth, OsNAC2 plays a critical role in rice response to abiotic stress (Mao *et al*., 2018; Shen *et al*., 2017). It is worth exploring how OsNAC2 integrates multiple phenotypes by targeting genes in different plant physiological processes. Plant transcriptional regulation is in precise combinatorial control of transcription factors, enhanceosome, holoenzyme, Pol II transcription‐initiation complex and transcriptional activators (Singh, 1998). For example, *Arabidopsis thaliana* WRKY33 interacts with transcription cofactor Mediator subunit16 (MED16) to activated transcription of PDF1.2 and ORA59 in necrotrophic fungal pathogen resistance (Wang *et al*., 2015). The phosphorylation, methylation or ubiquitination of transcription factors also lead to diverse function in plant development and environment response. The transcription factor Ideal Plant Architecture 1 (IPA1) activated *Dense and Erect Panicle 1* (*DEP1*) expression for higher yield, while phosphorylated IPA1 binds to the promoter of the pathogen defence gene *WRKY45* leading to enhanced disease resistance (Wang *et al*., 2018). Thus, we deduce that OsNAC2 may probably interact with other transcription cofactors, and the epigenetic modification of OsNAC2 may also occur in its regulation of multiple physiological process in plant. Yeast two‐hybrid and protein modification analysis should be performed to gain a further understanding of the regulatory mechanisms of OsNAC2.

## Experimental procedures

### Plant materials

The genetic background of rice (*Oryza sativa*) used in this study is Nipponbare. *OsNAC2* transgenic plants were the same as previously reported (Chen *et al*., 2015; Mao *et al*., 2017). For *OsNAC2* overexpression lines, the stop‐code‐less coding sequence of *OsNAC2* (Os04g0460600) was cloned into pCAMBIA1304 vector by *Nco*I and *Spe*I sites to generate the pCAMBIA1304‐OsNAC2. For *OsNAC2* RNAi lines, a specific 312‐bp coding sequence of *OsNAC2* was used as interfering fragment to construct RNAi vector. The sense and antisense of this specific fragment with a modified GUS intron were inserted into pHB vector and transformed into Nipponbare. The primers are shown in Table S1. Two vectors above and empty pCAMBIA1304 vector were introduced into rice callus by *Agrobacterium* strain EHA105. Homozygous transgenic lines were used for further analyses.

For targeted mutagenesis of *OsNAC2* using the CRISPR/Cas9 system, two gRNAs according to OsNAC2 coding sequence were chosen as cacggggcgcgctcccaag and ggacaaggacatcttcaga. gRNA was inserted into pBWA(V)H_cas9i2‐38720 vector and transformed into Zhonghua 11 (ZH11). The construction of recombinant plasmid and rice transformation were performed by Wuhan Biorun biological company. The identify primers are shown in Table S1.

Seeds with consistent germination were chosen to grow in the basal nutrient solution under a 16‐h light/8‐h dark photoperiod at 28°C. The length of primary roots and crown roots was measured in 2‐week‐old wild‐type and four *OsNAC2* transgenic lines, *ON7*,* ON11*,* RNAi25* and *RNAi31*. The number of crown roots was also counted.

### Hormone treatments

To determine the response of *OsNAC2* to different phytohormones, 1‐week‐old seedlings of WT were treated with 10 μm BA, 10 μm KT, 10 μm IAA, 10 μm 2,4‐D or 10 μm NAA. Total RNA of seedling roots was extracted 8 h after treatment and analysed by qPCR.

To analyse the effect of BA and IAA on *OsNAC2* transgenic plants, seeds were sterilized in 2% (w/v) sodium hypochlorite and then cultured aseptically for 6 d on the MS medium containing 2% (w/v) sucrose, 1% (w/v) agar (pH 5.8), IAA (0, 0.1 μm) and BA (0, 1 μm).

### β‐Glucuronidase staining

The root tips of 4‐ to 14‐day‐old transgenic plants expressing GUS driven by the *OsNAC2* native promoter were used to determine expression patterns of *OsNAC2*. For the IAA and cytokinin response experiment, 1‐week‐old transgenic *OsNAC2 pro*::GUS plants were treated with different concentration of IAA (0, 0.01, 0.1, 1 or 10 μm) and BA (0, 0.1, 1 or 10 μm) for 8 h, respectively. The examined tissues were soaked in 5‐bromo‐4‐chloro‐3‐indolyl‐β‐D‐glucuronic acid solution at 37°C overnight and then transferred to 70% ethanol to remove chlorophyll. Images were taken directly with a stereomicroscope (Leica ZOOM 2000, Leica Inc., Wetzlar, German).

### RNA *in situ* hybridization

The root tips of 5‐day‐old wild‐type plants were fixed in 4% (w/v) formaldehyde at 4°C overnight, and 6‐mm microtome sections were placed on RNase‐free glasses. The primers for probe preparation are shown in Table S1. The specific PCR fragment of *OsNAC2* amplified by PCR was inserted to pSPT19 through *Hind*III‐*Sac*I sites (Roche, www.roche.com). The RNA probes were then produced by T7 and SP6 polymerase labelled with digoxigenin according to the instructions (Roche DIG RNA Labeling Kit). *In situ* RNA hybridization and immunological detection were conducted according to Chen's description (Chen *et al*., 2015).

### Rice microarray analysis

For microarray analysis, total RNA was extracted from the roots of 2‐week‐old wild‐type and *OsNAC2* overexpression (*ON11*) plants grown in basal nutrient solution under a 16‐h light/8‐h dark photoperiod at 28°C. Biotin‐labelled cRNA was prepared using GeneChip 3′IVT Express Kit (Affymetrix) according to the manufacturer's instructions. The microarray process was conducted as previously described (Mao *et al*., 2017). The differentially expressed genes related to auxin and cytokinin in wild‐type and *ON11* plants were classified functionally using the biological process category of Rice Gene Ontology (www.geneontology.com).

### Quantitative PCR

Total RNA was extracted with RNAiso reagent (TaKaRa) from the leaves and roots of 2‐week‐old rice seedling. Purified total RNAs were reversed to first‐strand cDNA with PrimeScript RT Reagent Kit with gDNA Eraser (TaKaRa). qPCR analysis was performed using SYBR Premix EX Taq (TaKaRa) on MyiQ2 real‐time PCR detection system (Bio‐Rad, Hercules, California, USA) according to the manufacturer's instructions. Each sample was repeated three times for qPCR detection. *OsActin* was used as housekeeping gene for all analyses. The sequences of primers are listed in Table S1.

### Quantitation of free IAA and cytokinins

The root of 2‐week‐old *OsNAC2‐OX*, wild‐type and *OsNAC2‐RNAi* lines was collected and frozen in liquid nitrogen. For hormone content determination, the root fresh weight for each was sampled not <2 g. Extraction and quantification of the IAA content in each sample were performed as described previously (Fu *et al*., 2012). The endogenous cytokinins were quantified by using the polymer monolith microextraction/hydrophilic interaction chromatography/electrospray ionization tandem mass spectrometry method (Liu *et al*., 2010). Hormone content was measured at least in three biological repeats.

### The gravitropism experiments

To assess the root gravitropism response, *OsNAC2* transgenic and wild‐type seedlings were grown vertically for 3 days and then immediately relocated with 90° rotation. After 24 h, the root curvature of *OsNAC2* transgenic and WT seedlings was quantified.

### Amyloplast precipitation

One‐centimetre‐long root tips, excised from the 1‐week‐old *OsNAC2* transgenic seedlings, were soaked in 4% I_2_‐KI staining solution (8 g KI and 4 g I_2_) and stained for 5 min. After that, the samples were transferred to chloral hydrate solution (4 g chloral hydrate, 1 mL glycerol) to remove the unstable colour. Pictures were taken with a stereomicroscope (Leica ZOOM 2000; Leica Inc.)

### Chromatin immunoprecipitation (ChIP) sequence and ChIP‐qPCR

ChIP was performed based on the previous report (Gendrel *et al*., 2005) with *ON11* transgenic seedlings expressing OsNAC2‐mGFP fusion protein. Total protein was extracted from 2‐week‐old *ON11* transgenic seedlings grown in basal nutrient solution. At the same time, the wild type was used as the negative control. The OsNAC2 protein was immunoprecipitated using an antibody against GFP. The DNA fragments of the ChIP were used for sequencing and qPCR. The ChIP experiments were repeated three times with the similar data. Primer sequences for qPCR are listed inTable S1. For ChIP‐seq, libraries were generated using Ovation Ultralow Library System 2 (NuGEN) following the manufacturer's standard protocols. The total amount of the sample should be more than 20 ng. Sequencing was performed on a HiSeq 2000 (Illumina, San Diego, California, USA) using pair‐end 100‐bp mode. Peaks are referred to the regions of high sequencing read density. Integrative genomics viewer (IGV) was used to output a list of ‘peak calls’ that integrate with the genomic locations (Robinson *et al*., 2011).

### Yeast one‐hybrid and two‐hybrid assays

For yeast one‐hybrid assays, the coding sequence of OsNAC2 was inserted into *EcoR*I‐*Xho*I site of pGADT7 vector to generate a construct with activation domain and OsNAC2. Similarly, the promoter sequence of *OsGH3.6* (917 bp), *OsGH3.8* (801 bp), *OsARF25* (1493 bp) and *OsCKX4* (1120 bp) genes was inserted into pHIS2.1 vector through *Hin*dIII‐*Xho*I, *Sac*I‐*Mul*I, *Sac*I‐*Mul*I and *Sam*I‐*Xho*I sites to generate an in‐frame fusion with minHIS3. To test the interactions between OsNAC2 and OsRRs, yeast strain AH109 was transformed with pGADT7‐OsNAC2s and pGBKT7‐OsRRs. In case of self‐activation of OsRRs, AH109 was also transformed with pGADT7‐OsRRs and pGBKT7‐OsNAC2‐N’‐terminal region. All primers used for cloning these constructs are listed in Table S1. These constructed vectors and empty vector used as a negative control were transformed into yeast strain AH109 by the PEG/LiAc method (Gietz and Schiestl, 2007), and yeast cells were plated onto SD/‐His/‐Trp/‐Leu+50 mm 3‐amino‐1,2,4‐triazole (3AT) medium (Y1H) or SD/‐His/‐Trp/‐Leu/‐Ade (Y2H) for stringent screening of the possible interactions.

### EMSA

The coding region of OsNAC2 was amplified from rice genome and inserted into the pET‐28a vector (Novagen, Madison, Wisconsin, USA) through *BamH*I and *Xho*I sites. Primers are shown in Table S1. Protein expression and purification were carried out according to Yang's description (Yang *et al*., 2014). For preparation of fluorescence (FAM)‐labelled probes, FAM‐labelled oligos of the promoter regions of OsNAC2‐targeted gene, containing NAC binding site CACG, were synthesized by Sangon Biotech, and then, equal mole of paired oligos was mixed and annealed in 1×Taq DNA polymerase buffer from ToloBio.

Electrophoretic mobility shift assay was performed in a 20 μL reaction volume that contains 200 nmol probe and varied of OsNAC2 protein, in a reaction buffer of 50 mm Tris‐HCl [pH 8.0], 100 mm KCl, 2.5 mm MgCl_2_, 0.2 mm DTT, 2 μg salmon sperm DNA and 10% glycerol. After incubation for 20 min at 25°C, the reaction system was loaded into 10% PAGE gel buffered with 0.5×TBE. Gels were scanned with ImageQuant LAS 4000 mini (GE Healthcare, Boston, Massachusetts, USA ).

### Tilling mutant verification and hybridization with OsNAC2‐transgenic lines

The tilling mutants of *osgh3.6* and *osckx4* bought from Chunming Liu Laboratory at Key Laboratory of Plant Molecular Physiology, Chinese Academy of Agricultural Sciences. The mutants were grown in a standard paddy field at the Experimental Station of Fudan University in Taicang Jiangsu Province and grown under the conditions of conventional cultivation. For mutation verification, the leaf of 1‐week‐old mutant and wild‐type plants was used for DNA extract using the Plant Genomic DNA Kit (TIANGEN Code: DP305). The identification primers were provided by CAS, for amplifying the fragments of *OsGH3.6* and *OsCKX4*, respectively (Table S1). The obtained fragments were sequenced by Sangon Biotech (Shanghai, China). Additionally, total RNA was extracted with RNAiso reagent (TaKaRa, Dalian, China) from the roots of 2‐week‐old mutant lines, for identifying the genes related to auxin and cytokinin. For hybridization, *ON11* and *RNAi31* lines were used as female parents while homozygous *osgh3.6* and *osckx4* were used as pollen parent. The homozygote seeds from T2 generation identified by PCR (Figure S6) were used for further genetic phenotype analysis. The same primers were used for the identification of successful hybridization.

## Author contributions

F. M. conceived the project and Q.D designed the study; C. M. and J. H. performed the experiments; L. L., Y. Q. and P. L. provided technical assistance to C. M. and J. H.; C. M. (major part) and J. H. wrote the article; C. M. and F. M. revised the article; X. Y. and C. L. provide the tilling mutant. All of the authors discussed the results and commented on the manuscript.

## Conflict of interest

The authors declare that they have no conflict of interest.

## Supporting information


**Table S1** Primers used for the sequencing of different genes in rice.
**Table S2** List of cytokinin‐related genes altered in *ON11* roots (*P *< 0.05).
**Table S3** List of IAA‐related genes altered in *ON11* roots (*P *< 0.05).
**Table S4** List of cell cycling marker genes altered in *ON11* roots (*P *< 0.05).
**Figure S1** NAC binding motif CACG searching in positive fragments of *OsCKX4*,* OsARF25*,* OsGH3.6* and *OsGH3.8* promoters.
**Figure S2** Protein interaction between OsNAC2 and OsRRs.
**Figure S3** The expression of *OsCRL* and *OsCDK* genes 2‐week‐old WT and *OsNAC2* transgenic plants.
**Figure S4** Sequence alignment of mutant *osgh3‐6* with the wild type. wt stands for the wild type,while g19 and g20 are for different os*gh3.6* lines, *gh3.6‐1* and *gh3.6‐2*.
**Figure S5** Sequence alignment of mutant *osckx4* with wild type. wt stands for the wild type,while c2‐1 and c12‐1 are for two different *osckx4* lines*, osckx4‐1* and *osckx4‐2*.
**Figure S6** Identification of ON11*gh3.6 and RNAi31*ckx4 homozygote.
**Figure S7** Interaction between OsNAC2 and OsWOX11.
