# Supplementary material for: OsNAC2 integrates auxin and cytokinin pathways to modulate rice root development
Source: Plant Biotechnol J. 2019 Aug 7;18(2):429–42. doi: 10.1111/pbi.13209 (PMC6953191; doi:10.1111/pbi.13209)

**Supplemental Information**

**Table S1 Primers used for the sequencing of different genes in rice.**

| **Primer** | **Sequence** |
| --- | --- |
| **OsNAC2-Overexpression** |  |
| OsNAC2-F-*Nco*Ⅰ | TAccatggAGCAGCATCAGGGC |
| OsNAC2-R-*Spe*Ⅰ | CactagtGTAGCCCCATAGCGCG |
| **OsNAC2-RNAi** |  |
| *OsNAC2*-isenseF-*Hin*dⅢ/*Nco*Ⅰ | TTTaagcttCCATGGTCCTCCCTCCGCTGATG |
| *OsNAC2*-isenseR-*Eco*RⅠ | TAgaattcCGCTAAGCCTCTCCCGC |
| *OsNAC2*-iantiF-*Sac*Ⅰ/*Spe*Ⅰ | TTgagctcactagtTCCTCCCTCCGCTGATG |
| *OsNAC2*-iantiR-*Xba*Ⅰ | AAtctagaCGCTAAGCCTCTCCCGC |
| *OsNAC2-cas9-*ID-F | CTGGCTTCCGCTTCCACCCG |
| *OsNAC2-cas9-*ID-R | CAACACCCACTCGTTCTACGCACC |
| **In situ** |  |
| OsNAC2-pF-*Hind*Ⅲ | TTTaagcttGGCCGGAGGATG |
| OsNAC2-pF-*Sac*Ⅰ | TTgagctcGTCCAAGAACGAGTG |
| **OsNAC2-pET-28a** |  |
| OsNAC2-*BamH*I-F | CGggatccATGGAGCAGCATCAGGGCCAGGCA |
| OsNAC2- *Xho*I-R | CCGctcgagTTAGTAGCCCCATAGCGCGGCC |
| **qRT-PCR** |  |
| OsNAC2-F | GAGAAGTCTGGCTGGGTCAT |
| OsNAC2-R | AACACCCACTCGTTCTTGGA |
| OsActin-F | CTGCGATAATGGAACTGGT |
| OsActin-R | ACAATGCTGGGGAAGACA |
| OsGH3.1-F | GCAATGGAACAAAAGCAAGGA |
| OsGH3.1-R | CAGATCATCACCCTCTAGCTTCAA |
| OsGH3.6-F | CACTAGCATCTGTCTCATTGTGTCA |
| OsGH3.6-R | ACCTTGTCAGTGCCGGAATT |
| OsGH3.8-F | CGCGACGGTCCGAATAGT |
| OsGH3.8-R | GGAGGCATCCGGCTTCTACT |
| OsYUCCA5-F | ATGGTCGTCAGGGACAAGAT |
| OsYUCCA5-R | GGAACACTGAGAGGCCAAAT |
| OsYUCCA6-F | CCATTCCCAGATGGTTGGAAGG |
| OsYUCCA6-R | CATGTTGCGCCTCAAGATATTTG |
| OsCKX1-F | GCACCCGTGGCTCAACCTG |
| OsCKX1-R | GATGTCGGTGGCCGTCTGG |
| OsCKX4-F | TGTCAACCAACTGGAGATTGTG |
| OsCKX4-R | GAAGAGATCAGAGTTCACCTCGT |
| OsCKX5-F | CATCCTCATCTACCCCATGAA |
| OsCKX5-R | AACGCCACCAGGTAGAACA |
| OsCKX11-F | TGGCGAGATCTTCTACCTGGTG |
| OsCKX11-R | CAATGATTGCGTTGTTCTGCGCCA |
| OsLOGL3-F | GGAGGTGAGGCCAGTGTC |
| OsLOGL3-R | AGCAGCTCTTCCAGTGTTCC |
| OsIPT5-F | GCGGCTCCAACTCCTACA |
| OsIPT5-R | CAGGAAGCAGCAGTCGTACC |
| OsIPT3-F | GAGCTGTGCTTCCTGTGGGTGGACT |
| OsIPT3-R | GCGACCTTGTACTTGTCTCCGTGCG |
| OsRR1-F | CTCTGAAGCCGATCCCAGT |
| OsRR1-R | TCTTCAGCACCAGCATTCAT |
| OsRR3-F | GCAAGAGAGCACTGGAGGTT |
| OsRR3-R | ATCTCCGGCATGCAGTAATC |
| OsRR4-F | CATCCACCAAAGCCAAGAG |
| OsRR4-R | CAAATCGCCTCCATGATTTC |
| OsRR11-F | TTTCGTGTGACGGCTGTG |
| OsRR11-R | GGCATCCAGTAGTCCGTGAT |
| ORR2-F | CCCCCTCATTCTGATCCTTT |
| ORR2-R | GCTAGAGCAAGGTGAGTGAGG |
| ORR3-F | CGTCTGTAGCGATCGGTTG |
| ORR3-R | TCTGTCTGAATTTTAAGGGGTCA |
| OsIAA9-F | GGACCTGTGGTCACCTACGA |
| OsIAA9-R | CAGATCGTGCAAAATCATCC |
| OsIAA12-F | GCCATCACCTACCAGGACA |
| OsIAA12-R | TGATCCTCAACTTCTTGCATGT |
| OsIAA24-F | CATGCTTGTTGGAGATGTGC |
| OsIAA24-R | GCCCTTCATGATCCTCAACT |
| OsIAA3-F | GCCGTCACCTACGAGGAC |
| OsIAA3-R | TCCTCAACCTCTTGCACGTA |
| OsARF16-F | TGGATCACCCTGTAATGTCTCA |
| OsARF16-R | TGCTGCTGTTGTGATAGTTGG |
| OsARF25-F | CCACAACTTCTCTAACCAGG |
| OsARF25-R | TGTGGCTTGCAGAATAGTCG |
| OsPIN1b-F | TGCACCCTAGCATTCTCAGCA |
| OsPIN1b-R | CCCTCCTCCCAAATTCTACTTC |
| OsPIN1c-F | TCGCACGGGACGCAGTCA |
| OsPIN1c-R | CCCGTCCTTCTCGTTCTTGTTC |
| cdc2Os1-F | TCGCCGACCCTACCTACTC |
| cdc2Os1-R | CGTACTGCTCCATCAGGGTTA |
| cdc2Os2-F | TGCATGGTTTATGTTGAAACG |
| cdc2Os2-R | CTGGTAGAGGAACGACTGCAC |
| CDKB1;1-F | TCGGGATCGATGGAGAAGTA |
| CDKB1;1-R | GCCTTGTACACCTTCCCGTA |
| OsCRL1-F | AGCAACGTGTCCAAGCTGCT |
| OsCRL1-R | GTCCTGGTGGTGTATCCCTT |
| OsCRL4-F | CTGTGGAGCTTGATGAATACAC |
| OsCRL4-R | CAAGCTTCTCAGGCAACAAATG |
| OsCRL5-F | CCATCGACACGTTCGGTCAGAG |
| OsCRL5-R | GAGGTCCTTGTTGCCGGAGACT |
| OsWOX11-F | AACAGCGGCATGGTGAACCC |
| OsWOX11-R | GCCACGATGAGGACGACGAG |
| **Yeast One-Hybrid Assay** |  |
| OsGH3.1-EcoR I-F | CCGgaattcCGGTAATATATTAAGTACTA |
| OsGH3.1-Sac I-R | CgagctcGAAGATGTGTGAATGT |
| OsGH3.6-Hind III-F | CCCaagcttGGGTGGAGTGGTGCATATTAG |
| OsGH3.6 Xho I-R | CCGctcgagCGGTCCCGACCACCACGGGCG |
| OsGH3.8-Sac I-F | CgagctcGGGCTCGATCCGTCCTC |
| OsGH3.8-Mul I-R | CGacgcgtCGGGAACGCCTTCGAATG |
| OsCKX3-Hind III-F | CCCaagcttGGGTCATCCATGGCAAACATTTCAG |
| OsCKX3-Xho I-R | CCGctcgagCGGAAACAGCTGAAACAAACCCG |
| OsCKX4-Sam I-F | TCCcccgggGGACGGAGCAAATAAGACAGT |
| OsCKX4-Xho I-R | CCGctcgagCGGGGAGATTTAAAGGCCGGC |
| OsCKX8-Hind III-F | CCCaagcttGGGGACGGCACGAGATTGATCCA |
| OsCKX8-Sac I-R | CgagctcGCTTGCTCTAGCGTTGCAGTC |
| OsRR1-EcoR I-F | CCGgaattcCGGGGACATATTACTAGT |
| OsRR1-Sac I-R | CgagctcGGAGGGGAGGTGGGCCCA |
| OsRR3-Sac I-F | CgagctcGTTCTTGAATATTACTAGA |
| OsRR3-Mul I-R | CGacgcgtCGTTCTTGGCGATGGACAAG |
| OsRR6-Sac I-F | CgagctcGCCAAATATGGACTGACAC |
| OsRR6-Mul I-R | CGacgcgtCGAGGCGCAGCCATCGCTG |
| OsARF25-Sac I-F | CgagctcGTAAGCCATAGGGCAAAC |
| OsARF25-Mul I-R | CGacgcgtCGAACAAATCTGATGAAAAG |
| **ChIP-PCR** |  |
| OsGH3.6-F1 | TCGTCGCTCATGTTGTGCAAGC |
| OsGH3.6-R1 | ACCACCACGGGCGACGGCTGTC |
| OsGH3.6-F2 | GATATCCCAAGAGAGAGGAGGA |
| OsGH3.6-R2 | GGAGCTGCTCTAGGCGGCGTCG |
| OsGH3.6-F3 | TCGAGCTGATACCTAGGTATCA |
| OsGH3.6-R3 | ACGCTGATACCCAGGTACCAAG |
| OsGH3.6-F4 | GTTACTCTTGTTAGTCACGGGA |
| OsGH3.6-R4 | TACCCAAGGTACTAGGTATCAGT |
| OsGH3.6-F5 | TGGAGTGGTGCATATTAGGT |
| OsGH3.6-R5 | GGAATCCCGTGGGGTAGCAG |
| OsGH3.8-F1 | CCGGCCATTCGAAGGCGTTCCG |
| OsGH3.8-R1 | GTCATCACCGCCATTGCCTCTC |
| OsGH3.8-F2 | TTTTAAGTGTGAGATCCGACGT |
| OsGH3.8-R2 | GTATTTATAGAGGGCGAGGCG |
| OsGH3.8-F3 | TTGTTAATCGCGTCCGTTAGTC |
| OsGH3.8-R3 | CCGGCGGAGACGTGGCGGCTA |
| OsGH3.8-F4 | CGAAACGGACAGGCCATGTTC |
| OsGH3.8-R4 | TTACTAGTATCGGGCTTGAT |
| OsGH3.8-F5 | AGGCTTGCGCGCGAGAAAGTCG |
| OsGH3.8-R5 | TTTGCAAGGGGAGAGGGAGGAG |
| OsARF25-F1 | AAATGGAAGATATTTCTGGGCGCA |
| OsARF25-R1 | CTCCAATCACCAGTGCACCCGT |
| OsARF25-F2 | AGTTTTGGGTGAAAATACTCGAGC |
| OsARF25-R2 | TTTTCTCCCTCAACAACTAACC |
| OsARF25-F3 | GCTGTGTTTTGAGTGCTTGGGATG |
| OsARF25-R3 | GATGAAAAACACCCAGGCGACCGC |
| OsARF25-F4 | CGCGATCTCGTGAGCTCCGGGGG |
| OsARF25-R4 | ACCTTCACTCTCGGCGACGGCTA |
| OsARF25-F5 | GCACCTAGGGTTCGCGCCCCCAC |
| OsARF25-R5 | GAATCAGGCGACGACGACCACCA |
| OsARF25-F6 | TTTGTTTTACTACCTCCAAGAAG |
| OsARF25-R6 | TAGGGCTAGGCCGCCATGGCCGG |
| OsARF25-F7 | CATCCAAACAGCGTTTCCAAGGA |
| OsARF25-R7 | TCTGATGAAAAGAAAAAGGTCAG |
| OsARF25-F8 | TCAAGCCACACTTTATTAACACA |
| OsARF25-R8 | TTTGGTTGCGTGCCGCAGTTGAG |
| OsARF25-F9 | TTTTAAAATTTCCTATGAATTCA |
| OsARF25-R9 | CTCAACTGCGGCACGCAACCAAA |
| OsARF25-F10 | ACGGAGGGAGTACTATATTTGTC |
| OsARF25-R10 | TTATTGTTTGATTACACTAATAT |
| OsCKX4-F1 | AACACACGACATGGTGGTAC |
| OsCKX4-R1 | GGGAGGGAGATTTAAAGGCCG |
| OsCKX4-F2 | ACAGGTTCCAGGCGACTCCT |
| OsCKX4-R2 | TGTTAGCCCCAGTCTGCCAAA |
| OsCKX4-F3 | AATCCAATTTTATTCAGCACC |
| OsCKX4-R3 | GCCTGGTCTGCAAATTGCG |
| OsCKX4-F4 | TATGTTGCCATCAGGGAGATGCT |
| OsCKX4-R4 | GATCAACGTTTCGGTTAGCT |
| **Yeast Two-Hybrid Assay** |  |
| OsRR4-Sam I-F | TCCcccgggGGAATGACGGTGGTTGATGCGGAG |
| OsRR4-BamH I-R | CGCggatccGCGTCAGGTCTCCACTGCAAGGCC |
| OsRR6-Sam I-F | TCCcccgggGGAATGGCGGCAGCGGCGCAGGCT |
| OsRR6-BamH I-R | CGCggatccGCGTCATCTGATACGGCTGCAGAG |
| OsRR10-Sam I-F | TCCcccgggGGAATGGCAGTGGCTATAGAGGCT |
| OsRR10-BamH I-R | CGCggatccGCGTCAACTATGCCTTGGTCTTAT |
| OsNAC2-EcoR I-F | CCGgaattcCGGATGGAGCAGCATCAGGGC |
| OsNAC2’N-BamH I-R | CGCggatccGCGTTCTTGAACACCCTGCACAAC |

**Table S2 List of cytokinin-related genes altered in *ON11* roots (P<0.05)**

| Probe identifier | Fold Change | Gene | Annotation | References | Pathway |
| --- | --- | --- | --- | --- | --- |
| Os.14808.1.S1_at | 1.9157 | Os05g0311801 | *OsIPT3* | (Sakamoto et al., 2006) | CK biosynthesis |
| Os.16207.1.S1_at | 3.2309 | Os07g0211700 | *OsIPT5* | (Sakamoto et al., 2006) |  |
| Os.52793.1.S1_at | 1.2373 | Os04g0518800 | *OsLOGL6* | (Gu et al., 2015) |  |
| Os.11789.1.S1_at | 3.945 | Os03g0109300 | *OsLOGL3* | (Kurakawa et al., 2007) |  |
| Os.50470.1.S1_at | 0.4988 | Os01g0940000 | *OsCKX4* | (Gao et al., 2014) | CK oxidation |
| Os.33309.1.S1_at | 0.3341 | Os01g0775400 | *OsCKX5* | (Ashikari et al., 2005) |  |
| Os.46895.1.S1_at | 0.895 | Os10g0483500 | *OsCKX3* | (Ashikari et al., 2005) |  |
| Os.51112.1.S1_at | 0.6365 | Os03g0717700 | *OsHK4* | (Choi et al., 2012) | CK signaling |
| Os.8117.1.S1_at | 0.5309 | Os04g0442300 | *OsRR1* | (Kitomi et al., 2011a) |  |
| Os.24952.1.S1_at | 0.6192 | Os04g0673300 | *OsRR6* | (Hirose et al., 2007) |  |
| Os.24922.1.S1_at | 0.839 | Os02g0830200 | *OsRR3* | (Cheng et al., 2010) |  |
| Os.15908.1.S1_s_at | 0.6374 | Os01g0952500 | *OsRR4* | (Ito and Kurata, 2006) |  |
| Os.22367.1.S1_at | 0.5963 | Os07g0449700 | *OsRR7* | (Ito and Kurata, 2006) |  |
| Os.12448.1.S1_at | 0.2661 | Os02g0631700 | *OsRR11* | (Ito and Kurata, 2006) |  |
| Os.29407.3.S1_at | 0.5178 | Os08g0358800 | *OsRR13* | (Ito and Kurata, 2006) |  |
| Os.19406.1.S1_at | 1.742 | Os06g0183100 6g0183100 | *ORR2* | (Ito and Kurata, 2006) |  |
| Os.5243.1.S1_at | 1.5586 | Os02g0796500 | *ORR3* | (Ito and Kurata, 2006) |  |
| Os.27847.1.S1_at | 1.1749 | Os02g0182100 | *ORR4* | (Ito and Kurata, 2006) |  |

**Table S3 List of IAA-related genes altered in *ON11* roots (P<0.05)**

| Probe identifier | Fold change  (*P*<0.05) | Gene | Annotation | References | Pathway |
| --- | --- | --- | --- | --- | --- |
| Os.31989.1.S1_at | 0.4659 | Os12g0512000 | *OsYUCCA5* | (Yamamoto et al., 2007) | IAA biosynthesis |
| Os.5436.1.S1_at | 0.597 | Os07g0437000 | *OsYUCCA6* | (Yamamoto et al., 2007) |  |
| Os.52733.1.S1_at | 0.6753 | Os04g0128900 | *OsYUCCA7* | (Yamamoto et al., 2007) |  |
| Os.16010.1.S1_at | 1.5149 | Os05g0500900 | *OsGH3.4* | (Jain et al., 2006b) | IAA inactivation |
| Os.36449.1.S1_at | 1.2272 | Os01g0785400 | *OsGH3.1* | (Zhao et al., 2013) |  |
| Os.54708.1.S1_at | 1.5781 | Os05g0143800 | *OsGH3.6* | (Jain et al., 2006b) |  |
| Os.11798.1.S1_at | 1.4317 | Os07g0592600 | *OsGH3.8* | (Yadav et al., 2011) |  |
| Os.54625.1.S1_at | 0.9781 | Os12g0614600 | *OsPID* | (Morita and Kyozuka, 2007) | IAA transport |
| Os.2230.1.S1_at | 0.7041 | Os02g0743400 | *OsPIN1b* | (Wang et al., 2009) |  |
| Os.50938.1.S1_at | 0.8679 | Os11g0137000 | *OsPIN1c* | (Wang et al., 2009) |  |
| Os.54305.1.S1_at | 0.7825 | Os06g0232300 | *OsPIN1a* | (Xu et al., 2005) |  |
| Os.9945.1.S1_at | 0.4664 | Os03g0633800 | *OsIAA12* | (Jain et al., 2006a) | IAA signaling |
| Os.10109.1.S1_at | 0.517 | Os12g0601300 | *OsIAA30* | (Jain et al., 2006a) |  |
| Os.23256.1.S1_at | 0.4697 | Os02g0805100 | *OsIAA9* | (Jain et al., 2006a) |  |
| Os.7855.1.S1_at | 0.5264 | Os12g0601400 | *OsIAA3* | (Nakamura et al., 2006) |  |
| Os.49290.1.S1_at | 0.5527 | Os06g0597000 | *OsIAA23* | (Ni et al., 2011) |  |
| Os.8622.1.S1_at | 0.5861 | Os07g0182400 | *OsIAA24* | (Jain et al., 2006a) |  |
| Os.15798.1.S1 | 0.6441 | Os06g0166500 | *OsIAA20* | (Jain et al., 2006a) |  |
| Os.22917.1.S1_at | 0.689 | Os06g0196700 | *OsARF16* | (Shen et al., 2013) |  |
| Os.7177.2.S1_a_at | 0.5121 | Os04g0671900 | *OsARF12* | (Qi et al., 2012) |  |
| Os.22272.1.S1_at | 0.8947 | Os12g0613700 | *OsARF25* | (Qi et al., 2012) |  |
| Os.19756.1.S1_at | 0.8843 | Os03g0666100 | *OsCRL4* | (Liu et al., 2009) |  |
| Os.56321.1.S1_at | 0.8815 | Os07g0124700 | *OsCRL5* | (Kitomi et al., 2011a) |  |

**Table S4 List of cell cycling marker genes altered in *ON11* roots (P<0.05)**

| Probe identifier | Fold change  (*P*<0.05) | Gene | Annotation |
| --- | --- | --- | --- |
| Os.5060.1.S1_at | 0.586266 | Os08g0512600 | *CDKB2;1; cdc2Os3* |
| Os.11723.4.S1_x_at | 0.81702 | Os03g0118400 | *cdc2Os-1* |
| Os.3428.1.S1_x_at | 0.762324 | Os02g0123100 | *cdc2Os-2* |
| Os.12638.1.S1_at | 0.93313 | Os01g0897000 | *CDKB1;1* |
| Os.11347.1.S1_at | 0.592003 | Os01g0805600 | *CycB1;1* |
| Os.51953.1.S1_at | 0.726082 | Os12g0588800 | *CycD5;2* |
| Os.57113.1.S1_at | 0.570186 | Os12g0581800 | *CycA3;2* |
| Os.9590.1.S1_at | 0.603953 | Os12g0502300 | *CycA2;1* |
| Os.46852.1.S1_at | 0.511788 | Os10g0563900 | *CycP4;1; CYC U4;1* |
| Os.47947.1.A1_at | 0.895723 | Os08g0479300 | *CycD4;2* |
| Os.29475.2.S1_at | 0.448591 | Os08g0421100 | *CycD1;2* |
| Os.50582.1.S1_at | 0.718991 | Os07g0556000 | *CycD6;1* |
| Os.2693.1.S1_x_at | 0.501627 | Os06g0726800 | *CycB2;2* |
| Os.27595.1.S1_at | 0.547085 | Os06g0236600 | *CycD1;1* |
| Os.14184.1.S1_at | 0.688621 | Os06g0217900 | *CycD3;1* |
| Os.24881.1.A1_at | 0.525169 | Os05g0493500 | *CycB1;5* |
| Os.9246.1.S1_at | 0.626576 | Os04g0563700 | *CycB2;1* |
| Os.51142.1.S1_at | 0.731635 | Os04g0552300 | *CycP2;1* |
| Os.25463.1.S1_at | 0.715193 | Os03g0607600 | *CycA3;1* |
| Os.11708.1.S1_at | 0.706187 | Os03g0203800 | *CycD5;3* |
| Os.24649.1.S1_at | 0.406108 | Os02g0604600 | *CycF1;2* |
| Os.26853.1.A1_at | 0.79677 | Os02g0133000 | *CycT1;1* |
| Os.4195.1.S1_at | 0.8288 | Os01g0233500 | *CycA1;1* |

**Figure S1 NAC binding motif CACG searching in positive fragments of *OsCKX4*, *OsARF25*, *OsGH3.6* and *OsGH3.8* promoters.**

**
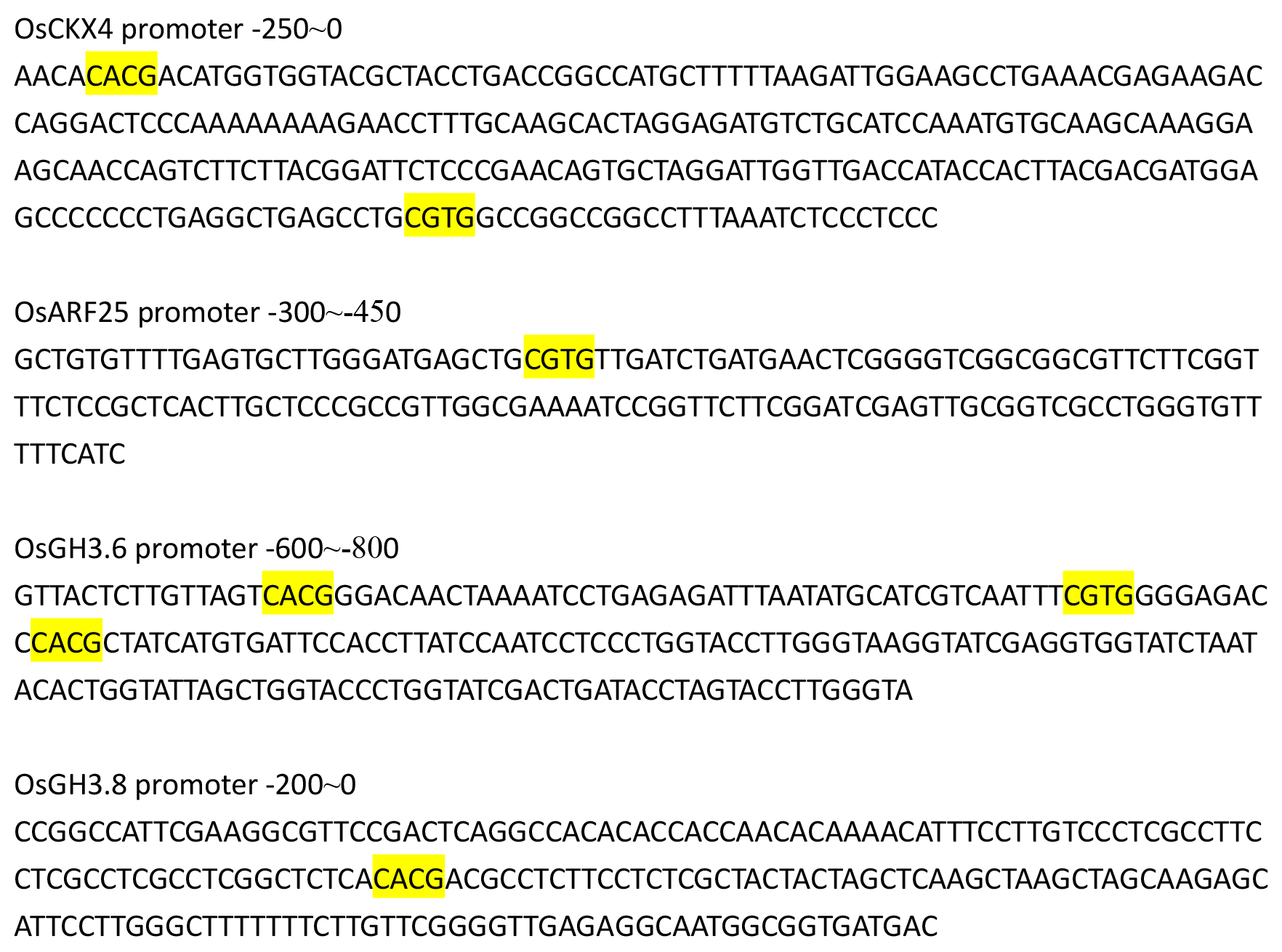
**

**Figure S2 Protein interaction between OsNAC2 and OsRRs.**

***
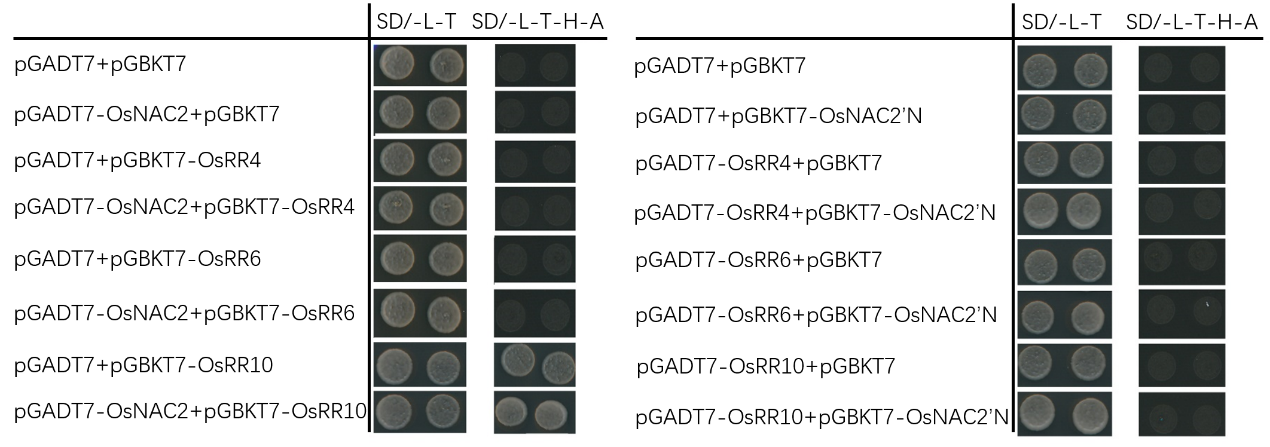
***

**Figure S3 The expression of *OsCRL* and *OsCDK* genes two-week-old WT and *OsNAC2* transgenic plants.** Asterisks represent statistically significant differences between WT and transgenic plants. **P*<0.05, ***P*<0.01, ****P*<0.001.

**
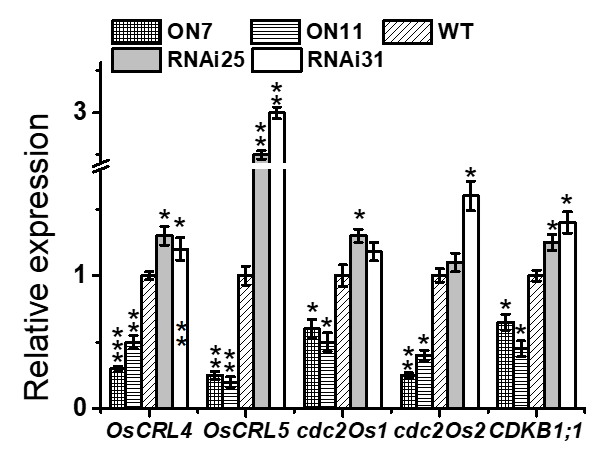
**

**Figure S4 Sequence alignment of mutant *osgh3-6* with the wild type.** wt stands for the wild type，while g19 and g20 are for different os*gh3.6* lines, *gh3.6-1* and *gh3.6-2.*


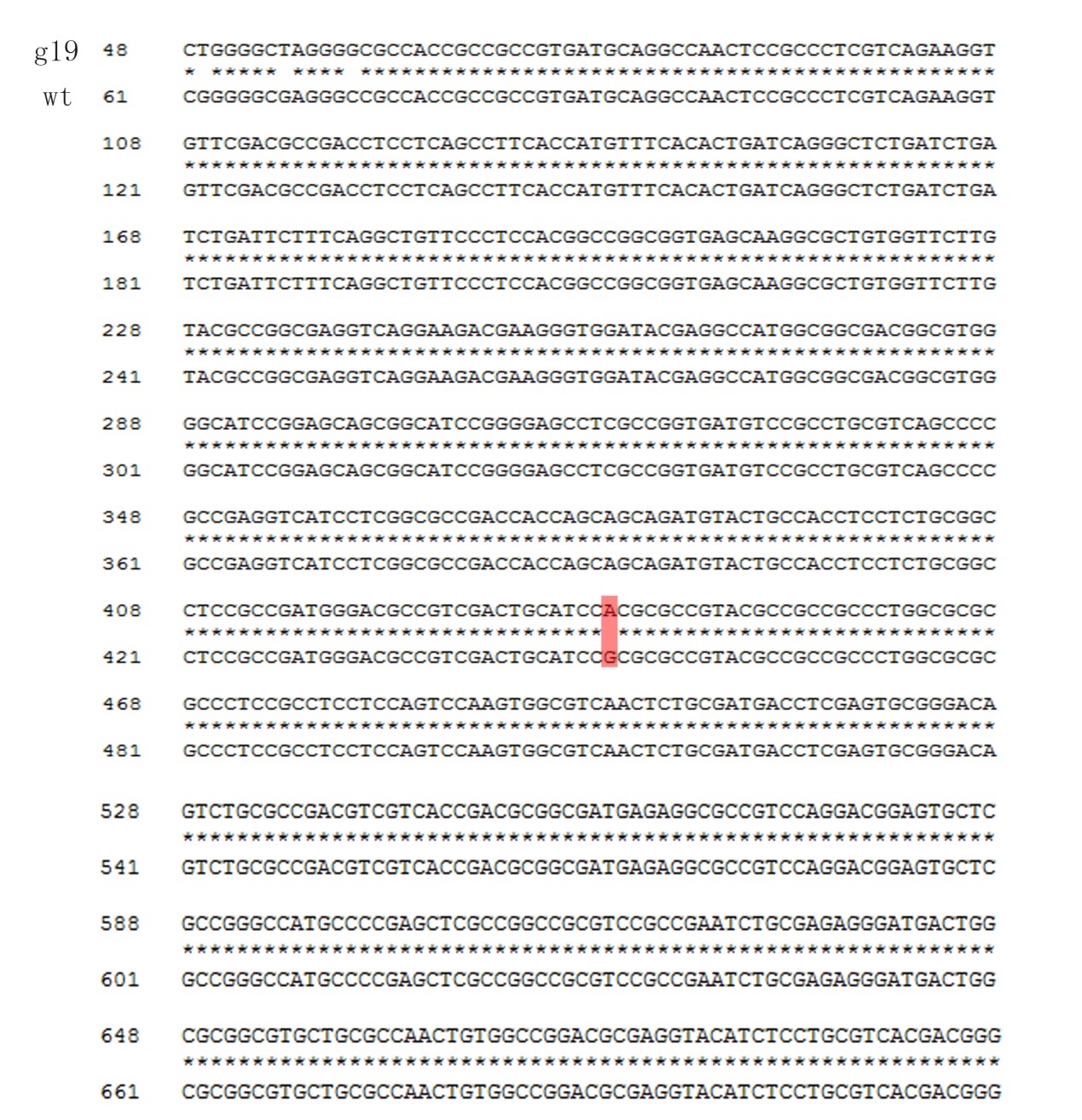


g20

\
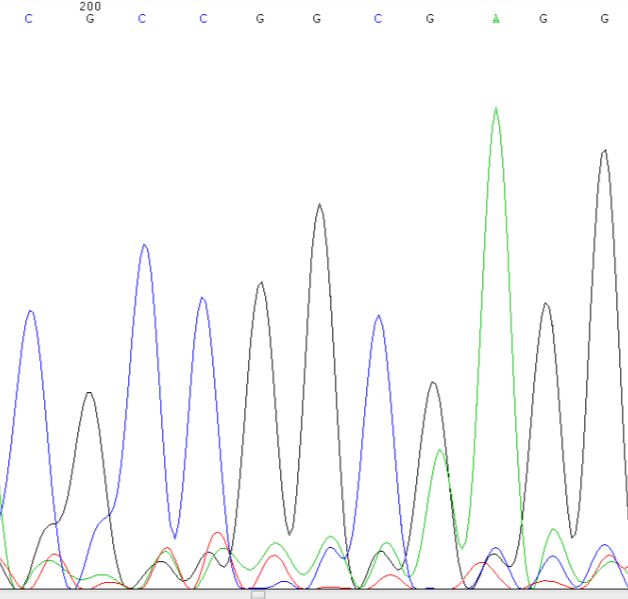


**Figure S5 Sequence alignment of mutant *osckx4* with the wild type.** wt stands for the wild type，while c2-1 and c12-1 are for two different *osckx4* lines*, osckx4-1* and *osckx4-2 .*


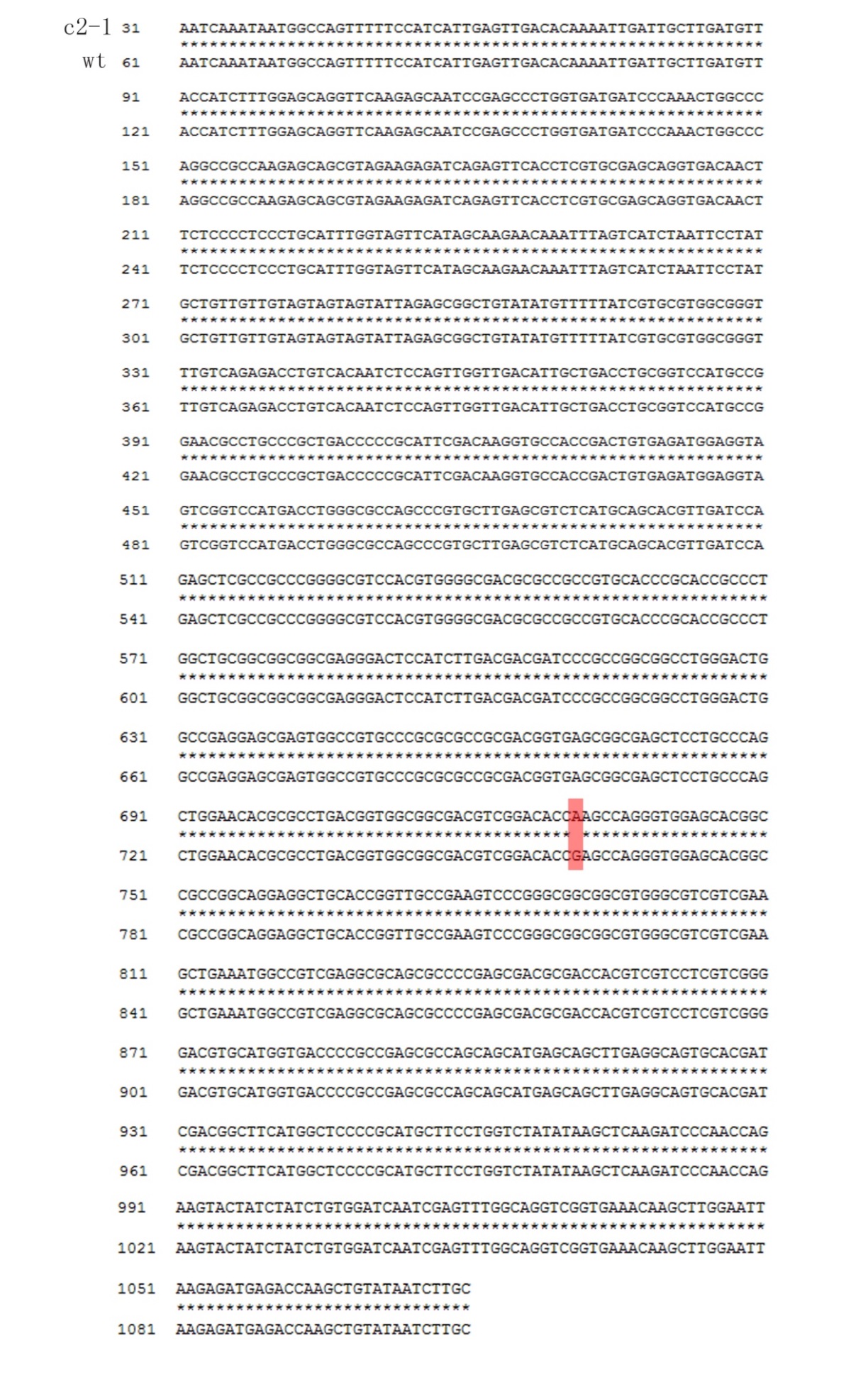

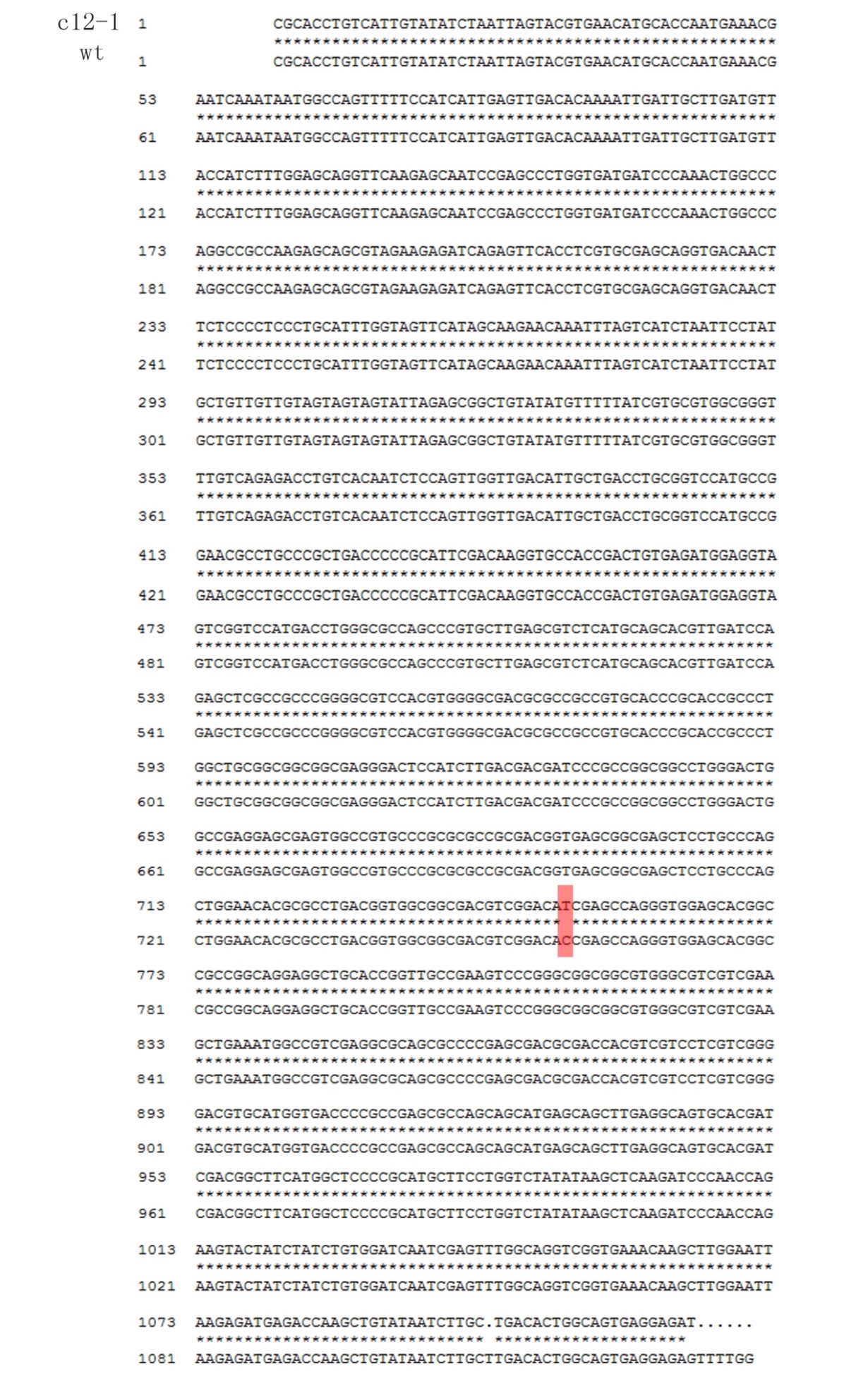


**Figure S6 Identification of *ON11*gh3.6* and *RNAi31*ckx4* homozygote.**

*ON11*gh3.6*


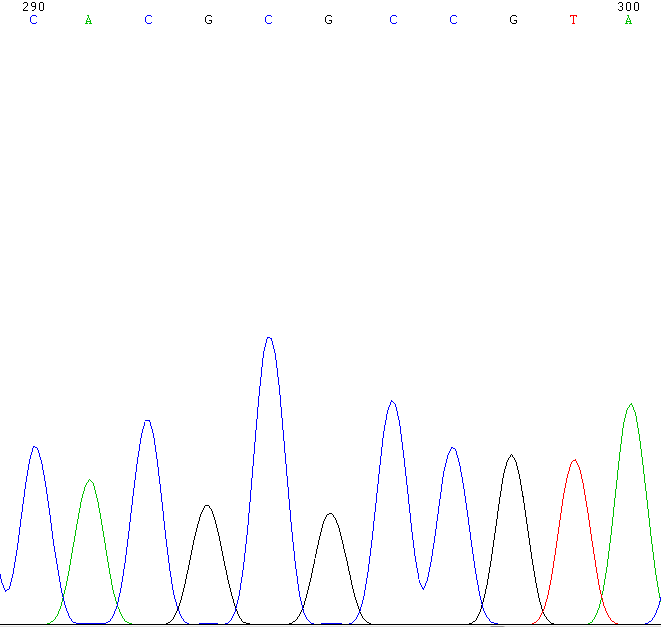


*RNAi31*ckx4*

*
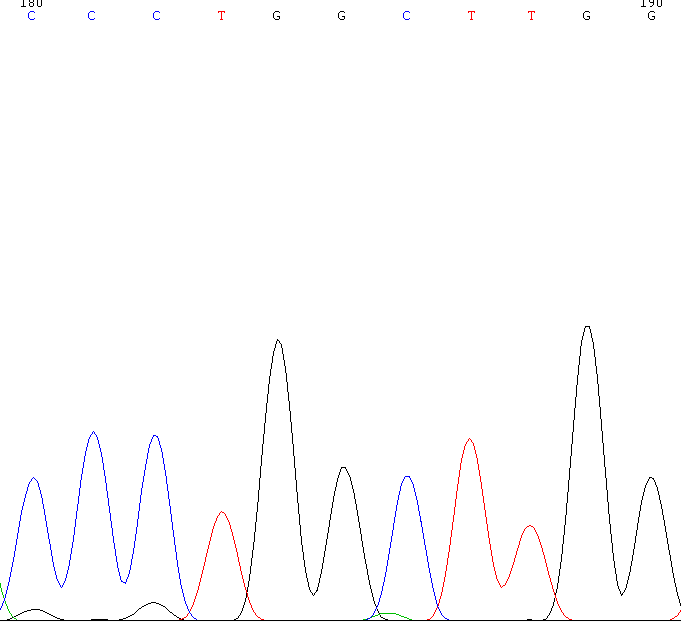
*

**Figure S7 Interaction between OsNAC2 and OsWOX11.**

**a,** Expression of *OsNAC2* in *oswox11* plant and *OsWOX11* in OsNAC2-OX and OsNAC2-RNAi lines. **b,** Yeast one-hybrid assays and yeast two-hybrid assays of the interaction between OsNAC2, OsWOX11 and their promoters.


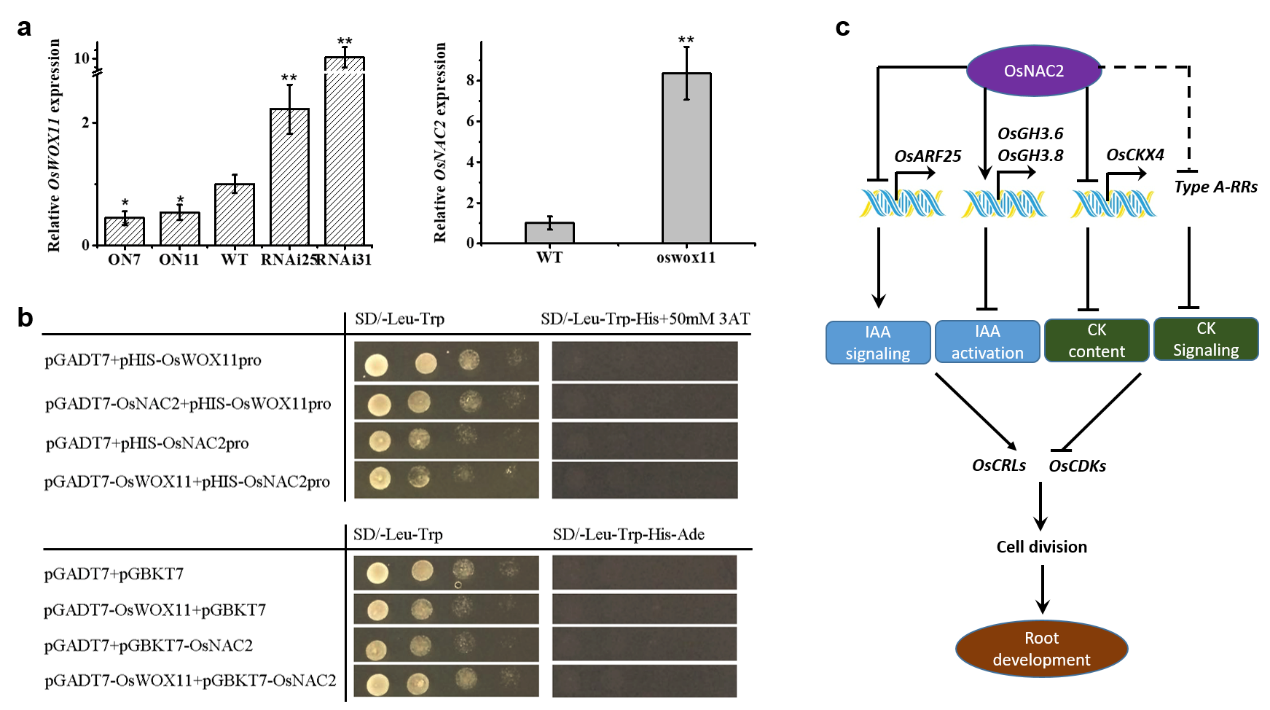

Supplement: Supplementary file 1 — Table S1 Primers used for the sequencing of different genes in rice. Table S2 List of cytokinin‐related genes altered in ON11 roots (P < 0.05). Table S3 List of IAA‐related genes altered in ON11 roots (P < 0.05). Table S4 List of cell cycling marker genes altered in ON11 roots (P < 0.05). Figure S1 NAC binding motif CACG searching in positive fragments of OsCKX4, OsARF25, OsGH3.6 and OsGH3.8 promoters. Figure S2 Protein interaction between OsNAC2 and OsRRs. Figure S3 The expression of OsCRL and OsCDK genes 2‐week‐old WT and OsNAC2 transgenic plants. Figure S4 Sequence alignment of mutant osgh3‐6 with the wild type. wt stands for the wild type,while g19 and g20 are for different osgh3.6 lines, gh3.6‐1 and gh3.6‐2. Figure S5 Sequence alignment of mutant osckx4 with wild type. wt stands for the wild type,while c2‐1 and c12‐1 are for two different osckx4 lines, osckx4‐1 and osckx4‐2. Figure S6 Identification of ON11*gh3.6 and RNAi31*ckx4 homozygote. Figure S7 Interaction between OsNAC2 and OsWOX11. [file PBI-18-429-s001.docx]
